# Epstein–Barr virus microRNA miR-BART2-5p accelerates nasopharyngeal carcinoma metastasis by suppressing RNase Ⅲ endonuclease DICER1

**DOI:** 10.1016/j.jbc.2023.105082

**Published:** 2023-07-24

**Authors:** Yangge Wu, Xiaoyue Zhang, Can Liu, Zhengshuo Li, Yuqing Wen, Run Zheng, Chenxiao Xu, Junrui Tian, Lingyu Wei, Jia Wang, Qun Yan, Xiang Zheng, Jian Ma

**Affiliations:** 1 NHC Key Laboratory of Carcinogenesis, Hunan Cancer Hospital and the Affiliated Cancer Hospital of Xiangya School of Medicine, Central South University, Changsha, China; 2 Cancer Research Institute and School of Basic Medical Science, Central South University, Changsha, China; 3 Key Laboratory of Carcinogenesis and Cancer Invasion of the Chinese Ministry of Education, Hunan Key Laboratory of Nonresolving Inflammation and Cancer, Changsha, China; 4 Department of Pathology and Immunology, Heping Hospital Affiliated to Changzhi Medical College, Changzhi, Shanxi, China; 5 Department of Clinical Laboratory, Xiangya Hospital, Central South University, Changsha, China; 6 Department of Pathology, Affiliated Hospital of Guilin Medical University, Guilin, Guangxi, China

**Keywords:** Epstein-Barr virus, miR-BART2-5p, DICER1, metastasis, nasopharyngeal carcinoma

## Abstract

The development and progression of nasopharyngeal carcinoma (NPC) is closely associated with Epstein-Barr virus (EBV) infection. NPC is usually asymptomatic until it spreads to other sites, and more than 70% of cases are classified as locally advanced disease at diagnosis. EBV-positive nasopharyngeal cancer tissues express only limited viral latent proteins, but express high levels of the EBV-encoded BamHI-A rightward transcript (BART) miRNA molecules. Here, we report that EBV-miRNA-BART2-5p (BART2-5p) promotes NPC cell invasion and metastasis *in vivo* and *in vitro* but has no effect on NPC cell proliferation and apoptosis. In addition, BART2-5p altered the mRNA and miRNA expression profiles of NPC cells. The development of human tumors has been reported to be associated with altered miRNAs expression, and overall miRNAs expression is reduced in many types of tumors. We found that BART2-5p downregulated the expression of several miRNAs that could exert oncogenic functions. Mechanistically, BART2-5p directly targets the RNase III endonuclease DICER1, inhibiting its function of cleaving double-stranded stem-loop RNA into short double-stranded RNA, which in turn causes altered expression of a series of key epithelial-mesenchymal transition molecules, and reverting DICER1 expression can rescue this phenotype. Furthermore, analysis from clinical samples showed a negative correlation between BART2-5p and DICER1 expression. According to our study, high expression of BART2-5p in tissues and plasma of patients with NPC is associated with poor prognosis. Our results suggest that, BART2-5p can accelerate NPC metastasis through modulating miRNA profiles which are mediated by DICER1, implying a novel role of EBV miRNAs in the pathogenesis of NPC.

Epstein-Barr virus (EBV) was the first human oncovirus to be identified and belongs to the gamma herpesvirus subfamily (1). About 95% of adults worldwide are healthy carriers of EBV (2). Almost all patients with nonkeratinizing nasopharyngeal carcinoma (NPC) are EBV-positive (3). In EBV-positive NPC, EBV infection is latent infection type II, expressing only EBV-encoded small RNA (EBER), EBV-associated nuclear antigen-1 (EBNA1), latent membrane protein 1/2 (LMP1 and LMP2), and BamHI-A rightward transcript (BART) miRNAs (4). In this state, EBV expresses very few proteins and is weakly immunogenic, but still highly expresses BART miRNA molecules, thus highlighting the importance of the latter. miRNAs are a class of noncoding single-stranded RNA molecules of approximately 22 nucleotides in length (5, 6), a structural feature that allows them to be relatively stable in the extracellular fluid, not readily degraded, and resistant to ribonucleases and extreme physicochemical conditions (*e.g.* pH, freezing, and heating), exhibiting many of its advantages as a potential minimally invasive cancer biomarker (7). Studies have shown that EBV-miRNA-BART2-5p (BART2-5p) presents a significantly higher level in the serum or plasma of patients with NPC, nasal natural killer/T-cell lymphoma and chronic active EBV infection compared to healthy controls (8, 9, 10, 11), suggesting that this virus miRNA molecule could be a new diagnostic marker or therapeutic target for NPC.

DICER1 is a highly conserved RNase III endoribonuclease (12). DICER1 performs its main biological functions by cleaving precursor miRNAs (pre-miRNAs) to produce 20 to 22 nt long mature regulatory miRNAs and siRNAs (13). It has been shown that DICER1 deficiency leads to miRNA dysfunction and promotes tumor progression (14). Meanwhile, DICER1 expression is also repressed by multiple miRNAs (15, 16, 17). In the current study, we discovered that BART2-5p accelerates NPC metastasis by suppressing DICER1. Moreover, BART2-5p can alter the overall mRNA profile and miRNA profile of the host cells. Since NPC is usually asymptomatic until it spreads to other sites, and more than 70% of cases are classified as metastasis disease at diagnosis, the prometastasis function of BART2-5p is worthy of notice.

## Results

### *EBV-miR-BART2-5p* promotes migration and invasion of NPC cells *in vitro*

**Figure 1:**
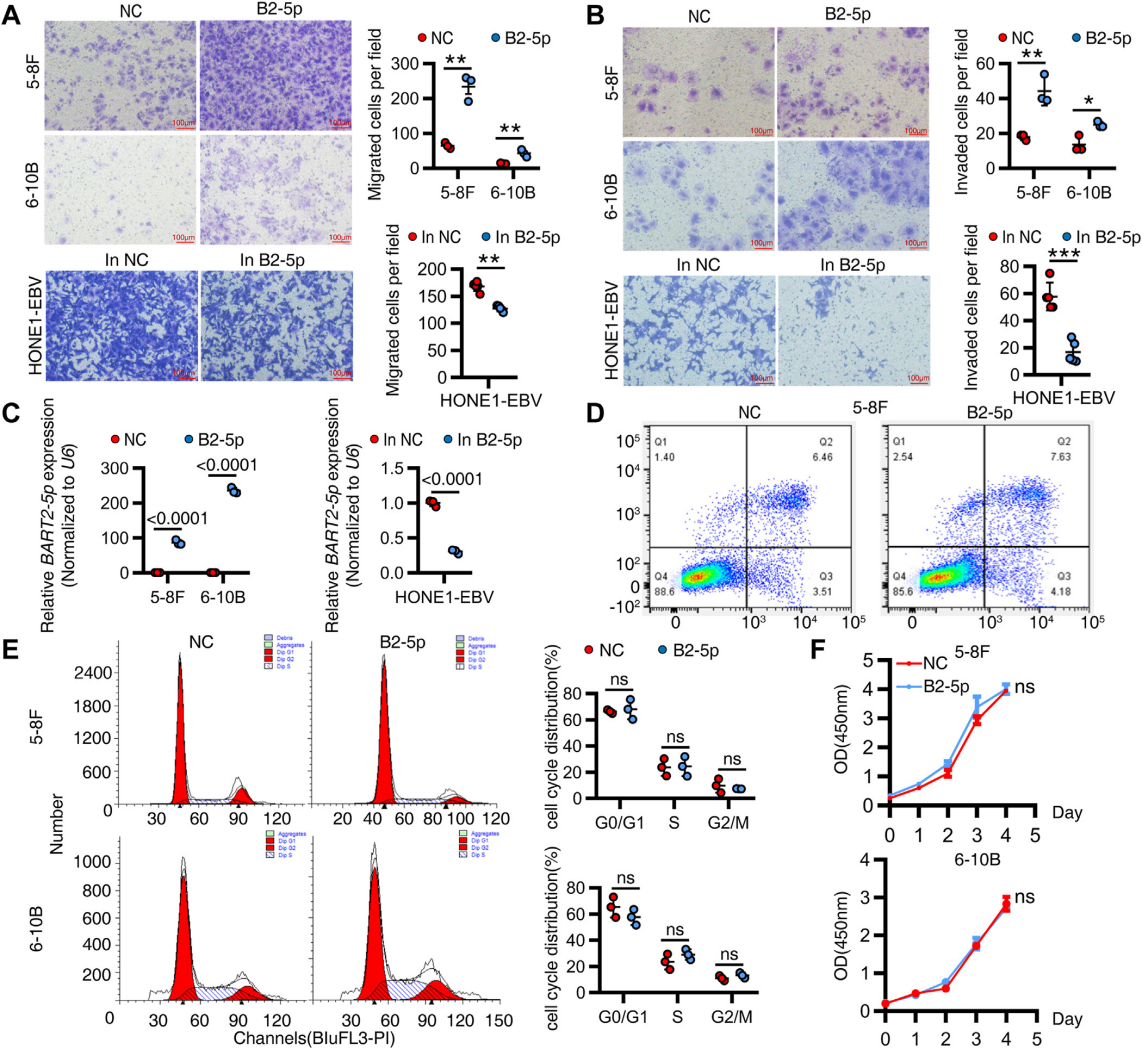
**BART2-5p promotes the migration and invasion of NPC cells *in vitro*.***A*, after transfection of BART2-5p mimics or NC (negative control) mimics into 5-8F and 6-10B cells for 48 h (n = 3, *upper panel*) or transfection of BART2-5p inhibitor and NC inhibitor into HONE1-EBV cells (n = 5, *bottom panel*), 30,000 cells from each group were inoculated in transwell chambers, and transwell migration assays were performed. *B*, thirty thousand NPC cells from each group were inoculated in transwell chambers, and transwell invasion assays were performed with representative images on the *left* and statistical plots of migrating cell numbers on the *right*. (*upper panel* for miRNA mimics and *bottom panel* for miRNA inhibitors). *C*, the transfection efficiency of miRNA mimics or inhibitors was detected by qRT-PCR. *D*, after transfection of BART2-5p mimics and NC mimics into 5-8F cells for 48 h, apoptosis was detected by flow cytometry. The representative images are shown. *E*, cell cycle was detected by flow cytometry. The representative image is shown on the *left*, and the statistical plot of the percentage of cells in G0/G1, S, and G2/M phases is shown on the *right* (n = 3). *F*, CCK8 assay to detect the proliferation of 5-8F cells and 6-10B cells transfected with BART2-5p or NC mimics (n = 5). Data are shown as the means ± S.D. Statistical significance relative to control was assessed by the unpaired two-tailed Student’s *t* test. ∗*p* < 0.05; ∗∗*p* < 0.01;∗∗∗*p* < 0.001; ns, *p* > 0.05 compared with the control group. (in NC, NC inhibitor; in B2-5p, BART2-5p inhibitor). BART, BamHI-A rightward transcript; NC, negative control; NPC, nasopharyngeal carcinoma; qRT-PCR, quantitative reverse transcription PCR.

**Figure 2:**
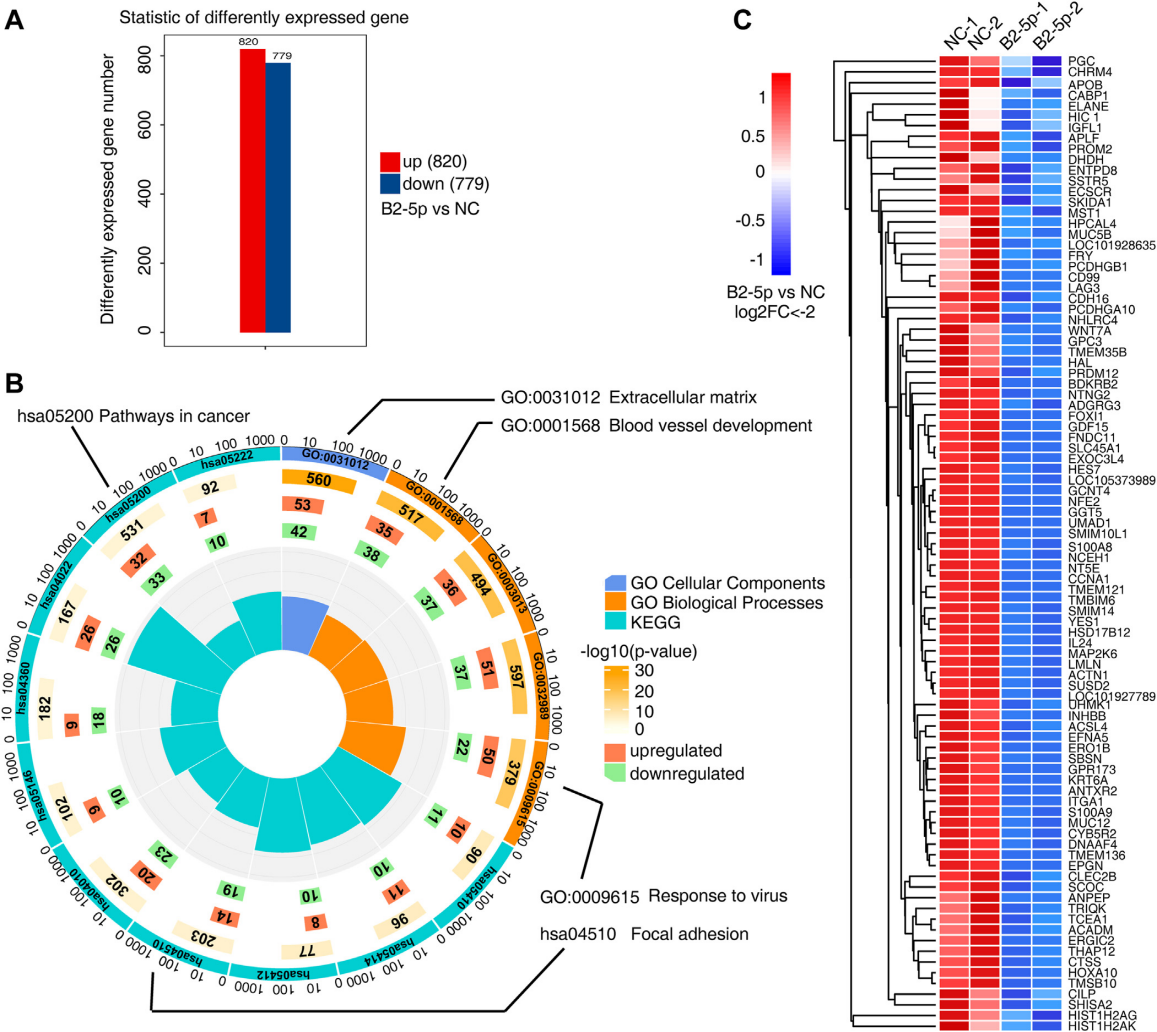
**BART2-5p modulates the mRNA expression profile of NPC cells.***A*, 6-10B cells were transfected with BART2-5p mimics or NC mimics for 48 h. RNA-seq was analyzed to compare the differentially expressed genes in the BART2-5p and NC cells, and the resulting differentially expressed genes were counted with *red* representing genes significantly upregulated in the BART2-5p group and *blue* representing genes significantly downregulated in the BART2-5p group. *B*, the differentially expressed gene (BART2-5p mimics *versus* NC mimics) screening criteria were set as *p* < 0.05, |log2FC|>1, and the obtained differential genes were subjected to KEGG and GO enrichment analysis. The top ten entries scored in the KEGG analysis and the top five entries scored in the GO analysis (scores are based on *p*-values) are shown in a *circle* chart. The *length of the rectangle* in the innermost *circle* indicates the number of genes contained in that entry, proportional to the ratio of the outermost *circle*. The *color of the rectangle* indicates the enrichment *p*-value (converted with -log10(p)) for that entry, with *darker colors* indicating smaller p. The second *green inner circle* indicates downregulated genes enriched to the pathway, and the third *orange circle* indicates upregulated genes enriched to the pathway. *C*, heatmap showing log2<−2 (4-fold downregulated) genes for RNA-seq (B2-5p mimics *versus* NC mimics). (4-fold upregulated genes are listed in Fig. S9). BART, BamHI-A rightward transcript; GO, Gene Ontology; KEGG, Kyoto Encyclopedia of Genes and Genomes; NC, negative control; NPC, nasopharyngeal carcinoma.

To investigate the effect of BART2-5p on NPC cells, we transfected BART2-5p mimics into EBV-negative NPC cells 5-8F and 6-10B cells, and transfected BART2-5p inhibitor into EBV-positive NPC cells HONE1-EBV as well. To our surprise, BART2-5p had no significant effect on apoptosis, cycle, and proliferation of NPC cells, but significantly promoted the migration and invasion abilities of them (Fig. 1, *A*–*F*). Similar phenomena were also noticed in normal nasopharyngeal epithelial cell line NP69 (Fig. S6, *A* and *B*). To gain a deeper and more comprehensive understanding of the molecular mechanisms by which BART2-5p promotes NPC migration and invasion, we transfected BART2-5p mimics into NPC cells 6-10B (Fig. S1*A*) and then analyzed the changes in gene expression profiles of the cells (Fig. 2). When setting the screening criteria at *p* < 0.05, |log2FC|>1, BART2-5p significantly upregulated 820 cellular genes and downregulated 779 genes compared to the control (negative control [NC] mimics) (Fig. 2*A*). We next subjected the differentially expressed genes (BART2-5p *versus* NC) to Kyoto Encyclopedia of Genes and Genomes analysis (Fig. 2*B*) and Gene Ontology (GO) analysis (Fig. 2*B*), and the enriched entries included focal adhesion, extracellular matrix, blood vessel development, and antiviral response. The results of these enrichment analyses suggest that BART2-5p affects the motility of NPC cells, and other processes, matching the results of our *in vitro* cellular assays (migration and invasion). We noted that DNA repair factor (APLF), forkhead box I1 (FOXI1), glucosaminyl (N-acetyl) transferase 4 (GCNT4), the transcriptional repressor Hes family BHLH transcription factor 7 (HES7), the tumor suppressor gene HIC ZBTB transcriptional repressor 1 (HIC1), and other important gene regulators were downregulated by BART2-5p (Fig. 2*C*). These gene list implies that BART2-5p′s function is quite complex and possibly is context-dependent.

### *EBV-miR-BART2-5p* affects the miRNA expression profile of NPC cells

**Figure 3:**
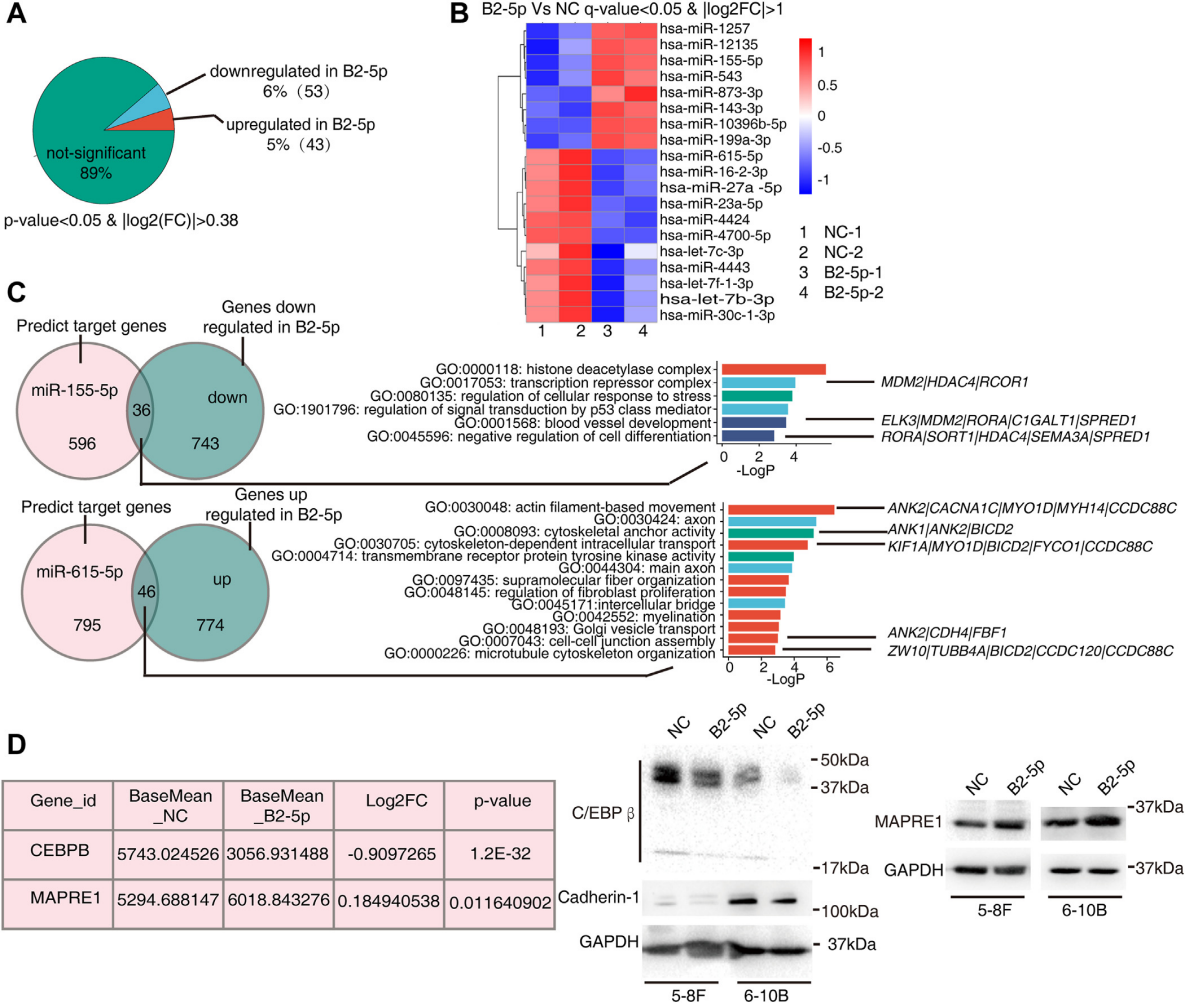
**BART2-5p modulates the miRNA expression profile of NPC cells.***A*, 6-10B cells were transfected with BART2-5p mimics or NC mimics for 48 h miRNA-seq was analyzed to compare the differentially expressed miRNAs in the BART2-5p and NC cells. Pie charts show the percentage of significantly differentially expressed miRNAs and not-significantly differentially expressed miRNAs in the total number of sequenced miRNAs (B2-5p mimics *versus* NC mimics). The screening criteria were set as *p* < 0.05, |log2(FC)|>0.38. *B*, the screening criteria were set as q < 0.05 and |log2FC|>1 and then the screened differentially expressed miRNAs (B2-5p mimics *versus* NC mimics) were heatmap clustered. *C*, the target genes of miR-155-5p and miR-615-5p were predicted using the bioinformatic tool ComiR (18), and the target genes with scores greater than 0.9 were intersected with the downregulated and upregulated genes from the mRNA sequencing data, respectively. The intersected genes were subjected to GO enrichment analysis, after which the genes enriched by the pathway of interest will be displayed. *D*, the expression of *CEBPB* and *MAPRE1* from the mRNA sequencing data were presented by a table (*left*). The protein levels of C/EBPβ, cadherin-1 and MAPRE1 were showed by Western blotting after transfection of BART2-5p mimics into 5-8F cells and 6-10B cells (*right*). BART, BamHI-A rightward transcript; GO, Gene Ontology; MAPRE1, microtubule-associated protein RP/EB family member 1; NC, negative control.

Further, we explored the effect of BART2-5p on the miRNA expression profile of NPC cells. We transfected BART2-5p mimics into NPC cells 6-10B, and then analyzed the changes of miRNA expression profiles of the cells (Fig. 3). Among the screened differentially expressed miRNAs, 53 were downregulated, accounting for 6% of the total number of miRNAs, and the number of upregulated miRNAs was 43, accounting for about 5% of the total number of miRNAs, and in general, downregulated miRNAs were slightly dominant in our system (Fig. 3*A*). After that, we set more stringent screening criteria q < 0.05 and |log2(FC)|>1, and screened 19 differentially expressed miRNAs for clustering for further study, among which 8 miRNAs were upregulated and 11 miRNAs were downregulated (Fig. 3*B*). Heatmap clustering showed that the miRNAs significantly downregulated in the BART2-5p group were hsa-let-7b-3p, hsa-let-7c-3p, hsa-let-7f-1-3p, hsa-miR-16-2-3p, hsa-miR-23a-5p, hsa-miR-27a-5p, hsa-miR-30c-1-3p, hsa-miR-4424, hsa-miR-4443, hsa-miR-4700-5p, and has-miR-615-5p; miRNAs that significantly upregulated in the BART2-5 group were hsa-miR-10396b-5p, hsa-miR-12135, hsa-miR-1257, hsa-miR-143-3p, hsa-miR-155-5p, hsa-miR-199a-3p, hsa-miR-543, and hsa-miR-873-3p (Fig. 3*B*). We next validated some of the significantly differentially expressed miRNAs with reverse transcription-quantitative PCR (RT-qPCR) experiments, and the validation results were basically consistent with the miRNA-seq results (Fig. S1*B*). The original expression-data files of mRNA-seq and miRNA-seq have been deposited at NCBI Gene Expression Omnibus under the accession number GSE220166.

BART2-5p significantly upregulated miR-155p-5p expression whereas downregulated miR-615-5p expression (Fig. 3*B*). We used the bioinformatics tool ComiR (18) to obtain potential target genes for significantly differentially expressed miRNAs with miR-155-5p and miR-615-5p as examples (Fig. 3*C*). Six hundred thirty-two target genes of miR-155-5p were identified (score >0.9). Among the 632 genes, 36 genes were downregulated in our mRNA-seq data (*i.e.* downregulated by BART2-5p expression), and these 36 downregulated genes can be enriched to transcription repressor complex and negative regulation of cell differentiation and other pathways. Eight hundred forty-one target genes of miR-615-5p were identified (score >0.9). Among the 841 genes, there are 46 genes that were upregulated in our mRNA-seq data (*i.e.* upregulated by BART2-5p expression), which are mostly related to cell motility and cell signaling, and suggested a possible impact of BART2-5p on cells motility.

To verify that BART2-5p indeed modulates those genes’ expression by influencing the production of miRNAs, we examined the mRNA and protein levels of CCAAT enhancer-binding protein β (C/EBPβ) and microtubule-associated protein RP/EB family member 1 (MAPRE1), molecules associated with epithelial-mesenchymal transition (EMT) events. C/EBPβ, a transcription factor, was validated as a target gene of miR-155-5p, which regulates the expression of molecules such as cadherin-1; MAPRE1 was validated as a target gene of miR-615-5p. Our results showed that miR-155-5p expression was upregulated after BART2-5p transfection (Fig. 3*B*), and its target gene C/EBPβ was repressed (Figs. 3*D* and S1*C*); whereas miR-165-5p expression was downregulated after BART2-5p transfection (Fig. 3*B*), and its target gene MAPRE1 expression was upregulated (Figs. 3*D* and S1*D*). These observations implied possible axis: BART2-5p—miR-155—C/EBPβ—cadherin-1; BART2-5p—DICER1—miR-615—MAPRE1.

### *EBV-miR-BART2-5p* directly targets DICER1 and inhibits its enzymatic function

**Figure 4:**
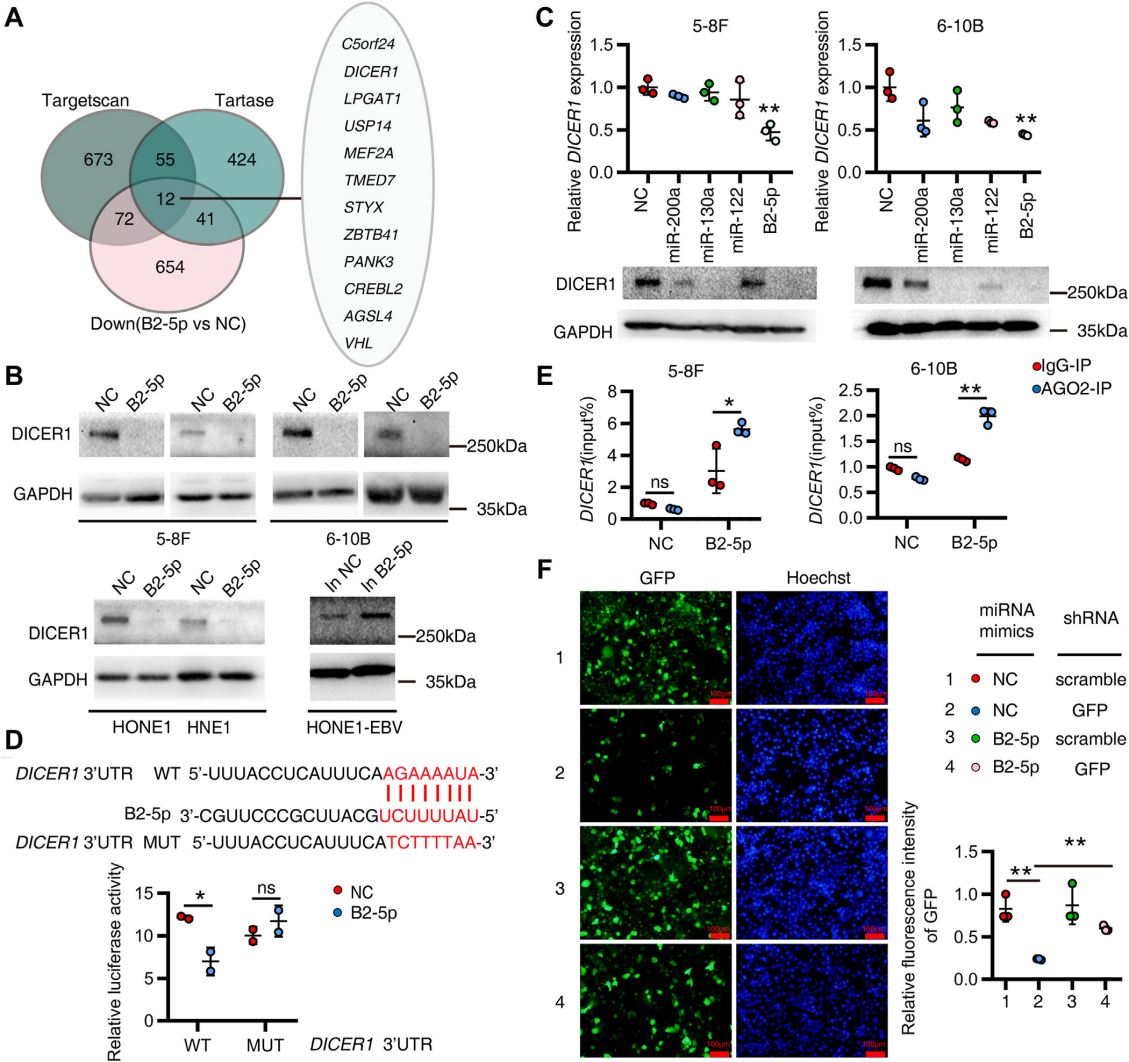
**BART2-5p directly targets DICER1 and inhibits its RNase endonuclease enzymatic function.***A*, the predicted target genes of B2-5p from two bioinformatics sites, TargetScan and TarBase, were intersected with genes significantly downregulated by B2-5p in RNA-seq results (B2-5p *versus* NC), and the Venn diagram showed that 12 genes were predicted to be BART2-5p target genes. *B*, BART2-5p mimics or NC mimics were transfected into multiple NPC cells, and BART2-5p inhibitor or NC inhibitor were transfected into HONE1-EBV cells. The protein levels of DICER1 were assayed. *C*, BART2-5p, miR-122, miR-200a, or miR-130a mimics were transfected into 5-8F and 6-10B cells, respectively. mRNA and protein levels of DICER1 were assayed. *D*, bioinformatic prediction of the binding site of BART2-5p to the DICER1 3′UTR region, showing WT and mutant (MUT) sequences (*upper panel*). Luciferase reporter gene assay using 293T cells to verify the direct BART2-5p-DICER1 3′UTR targeting relationship (*bottom panel*). The experiments were divided into four groups: (1) co-transfection of NC mimics and DICER1-luc-3′UTR-WT; (2) co-transfection of NC mimics and DICER1-luc-3′UTR-MUT; (3) co-transfection of BART2-5p mimics and DICER1-luc-3′UTR-WT; (4) co-transfection of BART2-5p mimics and DICER1-luc-3 ′UTR-MUT. The vertical axis indicates the relative luciferase activity values (n = 2). *E*, BART2-5p mimics or NC mimics were transfected into 5-8F cells and 6-10B cells for 48 h. Cell lysates were immunoprecipitated (IP) with anti-Ago2 antibody or negative control IgG. RT-qPCR was performed to detect the level of DICER1 mRNAs bound to Ago2 proteins (n = 3). *F*, characterization of the function of DICER1 RNase endonuclease cleaving stem-loop double-stranded RNA was performed using 5-8F cells stably expressing LV3-mock (H1/GFP & Puro), and experiments were divided into four groups: (1) co-transfection of NC mimics and scramble plasmids; (2) co-transfection of NC mimics and pLKO.1 GFP shRNA; (3) co-transfection of BART2-5p mimics and scramble plasmid; (4) co-transfection of BART2-5p mimics and pLKO.1 GFP shRNA. pLKO.1 GFP expression was observed under the microscope 48 h after transfection, representative images are shown on the *left*, and fluorescence intensity quantification is shown on the *right*. Ago2, Argonaute 2; BART, BamHI-A rightward transcript; NC, negative control; NPC, nasopharyngeal carcinoma; RT-qPCR, reverse transcription-quantitative PCR.

The classical way of binding between miRNA and its target mRNA is that the bases of seed region 2∼8 are fully complementary to each other. We thus input the seed sequence of BART2-5p 2∼8 "AUUUUCU" into Targetscan (19) to get the list of potential target genes of BART2-5p (673 potential target genes were revealed). To increase the reliability of the results and to narrow the scope of the study, we then entered the full sequence of BART2-5p "UAUUUUCUGCAUUCGCCCUUGC" into Tarbase (20), and also obtained a list of potential target genes of BART2-5p (424 potential target genes were revealed). After analyzing the RNA-seq results (Fig. 2*A*, GSE220166), we obtained a list of genes (654 genes) significantly downregulated by BART2-5p (*p* < 0.05 and log2FC<=-1). Finally, 12 potential target genes of BART2-5p were displayed in a Venn diagram, which included DICER1 (Fig. 4*A*). DICER1 is a very important molecule that regulates the synthesis and function of miRNAs, and it is a highly conserved RNase III endonuclease that generates 20∼22 nt long mature miRNAs by cleaving the precursor miRNAs (pre-miRNAs) and siRNAs to perform major biological functions. To investigate the effect of BART2-5p on DICER1, we transfected BART2-5p mimics into EBV-negative NPC cell lines 5-8F, 6-10B, HONE1, HNE1, and a normal nasopharyngeal epithelial cell line NP69, or transfected BART2-5p inhibitor into EBV-positive NPC cells HONE1-EBV, and analyzed the expression changes of DICER1. BART2-5p significantly downregulated the protein level (Fig. 4*B*) and mRNA level (Figs. S2 and S6*C*) of DICER1 in EBV-negative cells, and inhibition of BART2-5p upregulated the protein level (Fig. 4*B*) and mRNA level (Fig. S2*B*) of DICER1. It has been reported that miR-122, miR-200a, and miR-130a can suppress the expression of DICER1 (17, 21, 22). Among the four miRNAs, Bart2-5p′s suppression effect on endogenous DICER1 expression in NPC cells is the most significant (Fig. 4*C*). To clarify whether DICER1 is a direct target gene of BART2-5p, we found a binding site between BART2-5p and DICER1 3′UTR at position 2926 by bioinformatics prediction. We then cotransfected BART2-5p mimics with WT or mutant (MUT) luciferase reporter vectors of the corresponding site of DICER1 3′UTR in cells, respectively. Compared to control (NC mimics), BART2-5p mimics significantly decreased the luciferase activity of WT DICER1 3′UTR but not MUT (Fig. 4*D*). Argonaute 2 (Ago2) is a major component of the RNA-induced silencing complex , binding miRNAs and their mRNA targets in the ribonucleic acid complex. BART2-5p mimics significantly increased the level of DICER1 mRNAs bound to Ago2 (Fig. 4*E*), suggesting that BART2-5p directly targets the 3′UTR of DICER1 through RNA-induced silencing complex mechanism to inhibit its protein and mRNA levels. We then tested whether BART2-5p exerts an inhibitory effect on this function of DICER1 in cleaving stem-loop double-stranded RNA. We used 5-8F cells stably expressing GFP for characterization of DICER1 enzyme function, and GFP-shRNA was able to express stem-loop double-stranded RNA, which was subsequently cleaved by DICER1 into short siRNA to play a role in knocking down exogenous GFP expression (23). Our results indicate that BART2-5p significantly inhibited the function of GFP-shRNA, reflecting a blocked process of siRNA generation (Fig. 4*F*). And overexpression of DICER1 indiscriminately promoted the expression of EBV-encoded miRNAs (Fig. S3). Because pre-miRNA is contained within the sequence of the pri-miRNA, we detected the overall levels of pre-miRNA and pri-miRNA for miR-16-2-3p, miR-23a-5p, and let-7c-3p using real-time PCR with gene-specific primers as described (24, 25). We designed stem-loop primers to specifically reverse transcribe mature miRNAs, which were also detected by real-time PCR. Our experimental results showed that BART2-5p expression delayed the transition of pre-miRNA to mature-miRNA, which is a process modulated by DICER1 (Fig. S8).

We transfected BART2-5p mimics into EBV-positive cells HONE1-EBV in the first model (Fig. S7*A*). At first, the level of BART2-5p increased significantly, and then, it fell back over time, reaching a stable state after 84 h. During this process, *DICER1* mRNA levels were downregulated due to the elevation of BART2-5p, and there was a slight rebound (36 h–72 h) with the fall of BART2-5p, but it was still lower than the initial level. Similarly, we observed a slight increase in DICER1 protein levels at 48 h, 54 h, and 84 h after BART2-5p transfection comparing to 36 h (Fig. S7*D* right panel), taking into account the slight delay in the change in protein levels relative to the change in mRNA levels.

We used siRNA-DICER1 in the second model to reduce the expression levels of DICER1 in HONE1-EBV cells (Fig. S7*B*), during which we observed that the levels of BART2-5p were decreased due to the reduction of DICER1 (echoing Fig. S3*B* where overexpression of DICER1 can elevate BART2-5p levels), after exogenous siRNA-DICER1 depletion, there is a rebound in both BART2-5p and *DICER1* levels, with the rebound of BART2-5p (84 h) slightly delayed compared to *DICER1* (60 h), and our results suggested that BART2-5p can downregulate DICER1 mRNA and protein levels, while miRNAs generation controller DICER1 influences BART2-5p levels. These results suggest that BART2-5p directly targets DICER1 and inhibits its enzymatic function; meanwhile, DICER1 also can raise EBV miRNAs expression.

### *EBV-miR-BART2-5p* promotes invasion and metastasis of NPC cells through inhibition of DICER1

**Figure 5:**
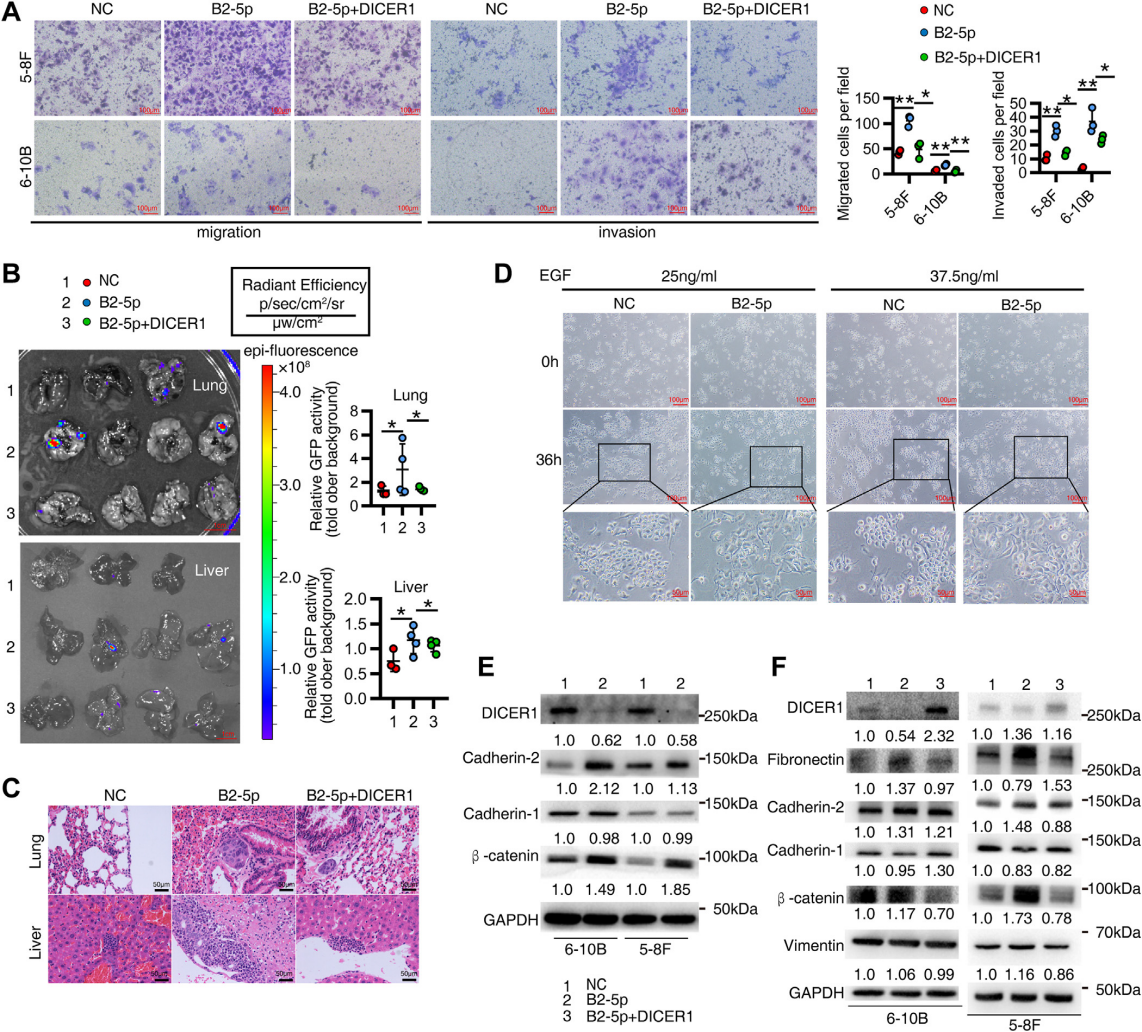
**BART2-5p promotes the invasion and metastasis of NPC cells by inhibiting DICER1.***A*, DICER1 expression plasmid and BART2-5p (or NC) mimics were co-transfected into 5-8F and 6-10B cells, transwell assay was performed to detect the migration and invasion abilities of NPC cells, and the representative images are shown in the *left panel*, and the statistics of the number of migrated and invaded cells are shown in the *right panel*. *B*, three groups of 6-10B NPC cells were injected into the tail vein of nude mice with 2 × 10^6^ cells per nude mouse, and the mice were executed 60 days later for GFP fluorescence imaging of lung tissue and liver tissue. The three groups of cells are as follows: NC control group (cells transfected with NC mimics), B2-5p group (cells transfected with BART2-5p mimics), and B2-5p+DICER1 group (cells transfected with B2-5p mimics and DICER1 expression plasmid), the *left panel* shows the imaging graph, and the *right panel* side shows the fluorescence intensity statistics. *C*, H&E staining demonstrating metastatic foci in lung and liver tissues of mice. *D*, 6-10B cells were employed for the morphological transformation experiment under the influence of enhanced green fluorescent protein. NC mimics and BART2-5p mimics were transfected into the cells for 24 h and EGF was added to stimulate the cells entering into EMT program. *E*, BART2-5p mimics or NC mimics were transfected into 5-8F cells and 6-10B cells, and Western blottings were performed to detect the protein levels of EMT-related markers. *F*, DICER1 expression plasmid and BART2-5p mimics were co-transfected into 5-8F and 6-10B cells, and Western blottings were performed to detect the protein expression of EMT-related molecules. BART, BART, BamHI-A rightward transcript; EGF, epidermal growth factor; EMT, epithelial-mesenchymal transition; NC, negative control; NPC, nasopharyngeal carcinoma.

Since DICER1 is a tumor suppressor gene and can inhibit tumor metastasis (26, 27), we asked whether BART2-5p promoted invasion and metastasis of NPC cells is through suppressing DICER1. Transwell assays showed that BART2-5p promoted the migration and invasion of NPC cells, and this phenomenon was reversed after reverting the expression of DICER1 (Fig. 5*A*). To investigate the role of BART2-5p in tumor metastasis, we constructed a tail vein injection tumor metastasis model in nude mice. Three groups of 6-10B cells (with GFP expression) were injected into the tail vein of nude mice. After 60 days, the mice were executed for GFP fluorescence biopsy, and liver and lung metastases were obtained. The three groups of cells were as follows: NC control group (6–10B cells transfected with NC mimics), B2-5p group (cells transfected with B2-5p mimics), and B2-5p+DICER1 group (cells transfected with B2-5p mimics and DICER1 expression plasmid). GFP *in vivo* imaging results showed that lung tissues (top) and liver tissues (bottom) of nude mice in the B2-5p experimental group had higher fluorescence intensity compared with NC control group and DICER1 reexpressing group (Fig. 5*B*). The surface nodules of lung and liver tissues showed more nodules on the surface of liver tissues (top) and more malignant metastases in lung tissues (bottom) in B2-5p group compared with the NC control group and DICER1 reexpressing group (Fig. S4). H&E staining of paraffin sections of liver and lung tissues from nude mice showed that the lung and liver metastases were more malignant in the B2-5p group compared with the NC control group and the DICER1 reexpressing group (Fig. 5*C*). Many studies have demonstrated that EMT plays a key role in tumor metastasis. So, we further investigated whether BART2-5p could alter the morphology of NPC cells and cause EMT. We found that the expression of BART2-5p significantly changed the morphology of NPC cells from subcircular to slender compared with NC control group, which is consistent with the morphological transformation of cells in EMT (Fig. 5*D*). We also examined the effect of BART2-5p on key EMT molecules and found that the expression of β-catenin, cadherin-2, and fibronectin were increased by BART2-5p, whereas cadherin-1 was slightly downregulated by BART2-5p (Fig. 5*E*). The changes in mRNA levels of multiple key EMT molecules after BART2-5p overexpression in the two NPC cell lines 6-10B and 5-8F were showed in Fig. S5*A*, DICER1 reexpression could reverse BART2-5p′s effect (Fig. S5*C*). These results suggest that BART2-5p can promote NPC cells metastasis, which is mediated by suppressing DICER1, at least partly.

### High expression of *BART2-5p* and low expression of DICER1 correlate with clinical malignant features of NPC

**Figure 6:**
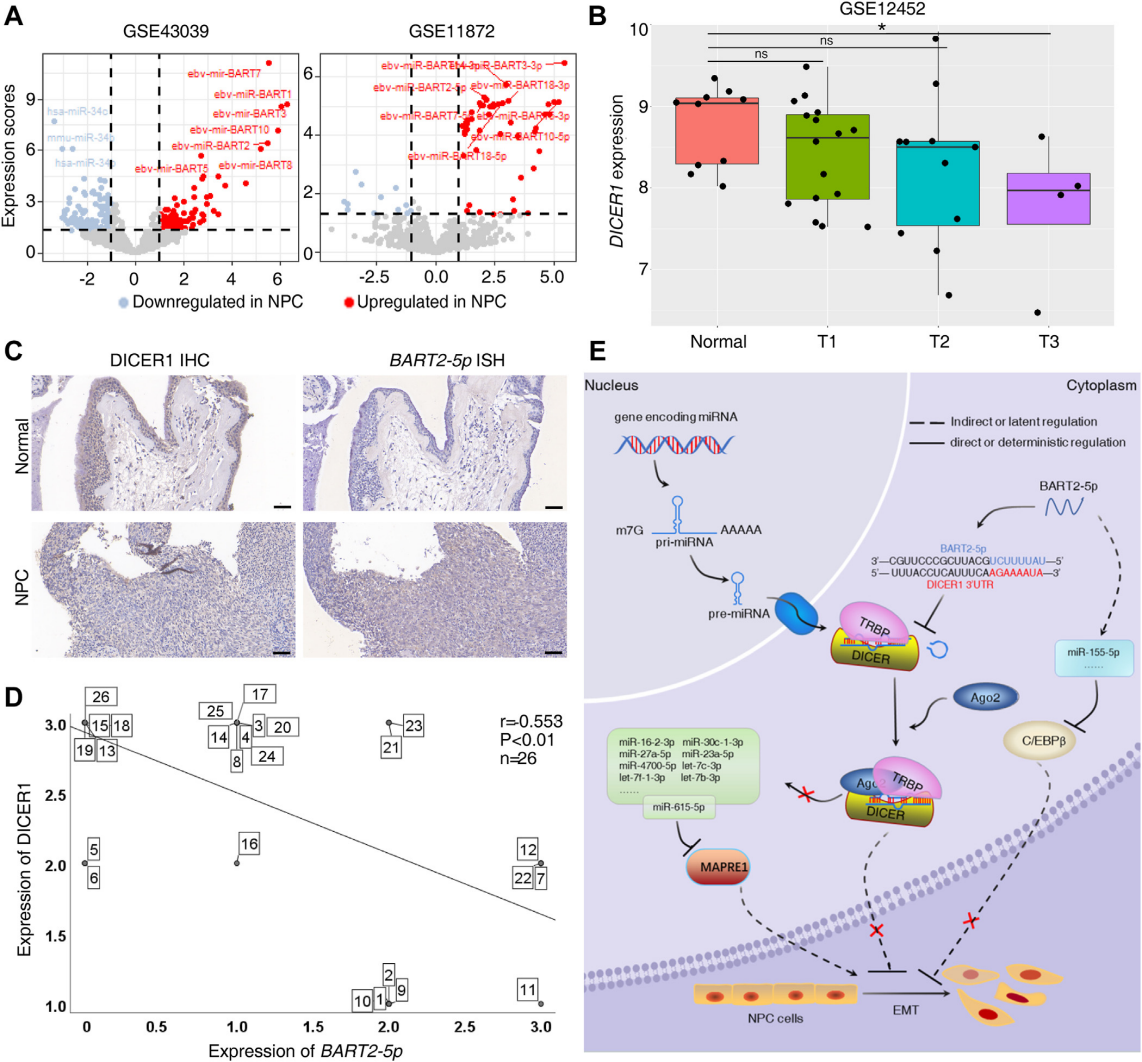
**High expression of BART2-5p and low expression of DICER1 were associated with clinical malignant phenotypes of NPC.***A*, comparing miRNA expression levels in NPC and chronic nasopharyngitis specimens (*left*, GSE43039); and in NPC biopsy specimens and normal nasopharyngeal mucosa specimens (*right*, GSE118720). *B*, *box line* plot showing the expression of DICER1 in NPC and normal nasopharyngeal mucosa specimens (GSE12452). Thirty-one NPC tissues in the tumor group were divided into three groups according to the T stage of the patients: group T1 (n = 16), group T2 (n = 11), and group T3 (n = 4); the horizontal axis indicates the grouping and the vertical axis is the expression level of DICER1. *C*, DICER1 and BART2-5p were detected in NPC tissue specimens and in normal nasopharyngeal epithelial tissue by means of immunohistochemistry and RNA *in situ* hybridization staining, respectively. *D*, DICER1 levels were detected in eight normal nasopharyngeal epithelial tissues and 18 NPC samples by immunohistochemistry; BART2-5p levels were detected by RNA *in situ* hybridization. According to the intensity and amount of cell staining, they were classified into four levels, with 0 indicating no positive staining (negative), one indicating *light yellow* (weakly positive), two indicating *brown* (positive), and three indicating *brown* (strongly positive), and finally, correlation plots were drawn, with the vertical axis representing DICER1 expression levels and the horizontal axis representing BART2-5p expression levels, and Spearman's correlation coefficient r = −0.553, *p* < 0.01. The number in the box indicates the sample number, there are 26 cases. *E*, a schematic diagram illustrating the proposed role of *BART2-5p* in promoting NPC metastasis by inhibiting DICER1. BART, BamHI-A rightward transcript; NPC, nasopharyngeal carcinoma.

Through analysis of NPC clinical specimens mRNA and miRNA expression profiles (GSE43039 and GSE118720) (28, 29), we noticed that EBV miRNA-BARTs were significantly highly expressed in NPC tumor tissues, such as BART2-5p, BART3-3p, BART8, BART7-5p, BART10-5p, and so on., while host cell-encoded miRNA molecules showed low expression, such as has-miR-34c, has-miR-34b, and so on (Fig. 6*A*). We further investigated the correlation between DICER1 and the development of NPC by analyzing the NPC gene expression profile data, GSE12452 (30), which was measured by Affymetrix Human Genome U133 Plus 2.0 array, from ten normal nasopharyngeal tissues and 31 nasopharyngeal cancer tissues. We divided the 31 nasopharyngeal cancer tissues into T1, T2, and T3 groups according to the tumor T-stage. As shown in the box plot, compared with the normal nasopharyngeal group, the expression levels of DICER1 in the T3 group was significantly lower and statistically different, *p* < 0.05; while the expression values of DICER1 in T1 and T2 stages were not significantly different from those in the normal control group, the median expression level of DICER1 in patients with NPC at all stages was lower than that in the normal control group. DICER1 is expected to be a potential target gene for BART2-5p (Fig. 4*D*). We collected 18 samples from NPC patients and eight samples from patients with nasopharyngeal polyps and detected a negative correlation between DICER1 and BART2-5p expression in these clinical samples (Fig. 6*C*), which was statistically significant (Fig. 6*D*).

In summary, we revealed that EBV-encoded miRNA BART2-5p is a prometastasis miRNA, and one of its target gene is DICER1, a master regulator of miRNA maturation and function. BART2-5p can significantly promote the expression of oncogenic miRNAs such as has-miR-155-5p (31, 32) and hsa-miR-543 (33), and also hinder the production of tumor-suppressor miRNAs such as hsa-let-7b-3p (34), hsa-let-7c-3p (35), and hsa-miR-615-5p (36), which eventually led to the occurrence of EMT in NPC cells and promoted the invasion and metastasis of NPC cells (Fig. 6*E*).

## Discussion

EBV infection is associated with many diseases, including NPC, gastric cancer, Hodgkin's lymphoma, systemic lupus erythematosus, and multiple sclerosis (37, 38). Most EBV-associated tumors of epithelial cell origin belong to EBV latent infection type I or II. These tumor cells express few viral proteins and are weakly immunogenic, but highly express viral BART miRNAs. BART miRNAs are derived from the EBV-encoded BART (39). Their abundance in tumor cells suggests that they may be a major factor in EBV-influenced tumorigenesis. BART miRNAs have been shown to regulate multiple genes with powerful functions, such as BART1-3p, BART1-5p, and BART7-3p to directly target the cellular tumor suppressor phosphatase and tensin homolog deleted on chromosome ten, thereby promoting NPC cell metastasis (28, 40). We previously reported that BART3-3p directly targets the coding DNA sequence region of TP53 to promote proliferation and inhibit cellular senescence in gastric cancer cells (41); BART5-3p can also target the 3′-UTR region of TP53, leading to accelerated cell cycle progression and inhibition of apoptosis, and so on (42). BART miRNAs have been shown to have potential roles involving cancer growth, tumor invasion, evasion of host immune surveillance, and lipid metabolism. For example, BART1-3p can target and inhibit E2F3 thereby inducing cell cycle arrest and inhibiting cell growth in gastric cancer cells (43); BART13 promotes NPC cell growth and metastasis by targeting the NKIRAS2/NF-κB pathway and thereby promoting NPC cell growth and metastasis (44); BART8-3p targets the inhibition of RNF38 to promote NPC metastasis (29); BART6-3p inhibits EBV-triggered interferons-β response and promotes EBV infection by targeting the 3′UTR of retinoic acid-inducible gene I mRNA (45); BART18-3p can regulate *de novo* adipogenesis to promote colorectal cancer progression, and so on (46). Previous studies have shown that BART2-5p can target and inhibit Rho family GTPase 3 and activate Rho signaling to enhance tumor cell motility (47). In contrast, we found that BART2-5p can directly target and inhibit DICER1 to trigger EMT in NPC cells and promote NPC cell invasion and metastasis, elucidating the molecular mechanism of NPC metastasis from another perspective.

In recent years, BART miRNAs have gained increasing attention as biomarkers by virtue of their miRNA structural advantages. Studies have shown that BART miRNAs can be released from EBV-infected cells into the bloodstream, loaded in exosomes or otherwise transferred from malignant to nonmalignant cells (10, 48, 49). For example, EBV-positive gastric cancer cells secrete BART15-3p *via* exosomes, a molecule that targets BRUCE to induce apoptosis in recipient immune cells (50). In patients with EBV-related diseases, the levels of some BART miRNAs are significantly higher and stably present in serum or plasma than in healthy controls. As an example, serum levels of BART2-5p can successfully distinguish patients with nasal natural killer/T-cell lymphoma from healthy controls (8, 51). Patients with NPC have high levels of BART2-5p in both tissues and serum (9, 52). Both primary and recurrent NPC have significantly higher BART2-5p levels in plasma compared to healthy controls (53); plasma BART2-5p levels were significantly higher in patients with chronic active EBV infection than in patients with infectious mononucleosis and healthy controls; plasma BART2-5p levels were significantly higher in patients with chronic active EBV infection with systemic symptoms compared to patients without systemic symptoms, meaning that BART2-5p expression levels were higher in patients with more severe disease (10). In addition, we found that BART2-5p significantly promoted NPC cell invasion, migration, and metastasis, while having no effect on cell proliferation or apoptosis, which aroused great interest. This phenomenon was partially explained in the subsequent mechanistic studies: focal adhesion, extracellular matrix, regulation of cell adhesion, and pathways in cancer, which are closely related to tumor metastasis, are among the top 10 or 5 of BART2-5p-regulated NPC signaling pathways (Fig. 2*B*). BART2-5p also regulates miRNAs expression profiles in host cells, affecting extracellular matrix, tube morphogenesis, axon, and other pathways (Fig. 3*C*). In contrast, proliferation-related pathways and apoptosis-related pathways accounted only for a smaller proportion of the enriched signaling pathways. Therefore, we hypothesized that BART2-5p has a biased function, which is to promote the metastasis of EBV-associated tumors. A meta-analysis of 46 studies on 20 different types of human cancer revealed that decreased expression of let-7 family miRNAs and increased expression of the cancer-promoting miR-21 were most commonly associated with poor prognosis (54). We note that let-7 family miRNAs such as hsa-let-7b-3p, hsa-let-7c-3p, and let-7f-1-3p are significantly repressed by BART2-5p. The expression level of miR-155-5p was highly correlated with the prognosis of patients with metastatic melanoma (55), and miR-155-5p could be significantly increased by BART2-5p. Metastasis is an important cause of NPC recurrence, poor prognosis, and cancer-related death and is also an urgent problem to be solved. Therefore, relevant studies on BART2-5p may provide new treatment strategies for NPC patients with recurrence and poor prognosis.

We found that BART2-5p regulates multiple genes’ expression, including DICER1, and that DICER1 is a direct target gene of BART2-5p. DICER1 is a highly conserved RNase III endoribonuclease, which is a key enzyme for cleaving precursor miRNA (pre-miRNA) to produce 20 to 22 nt long mature miRNA and short interfering RNA. In addition, DICER1 can function as an antiviral through RNase endonuclease (56). DICER1 damage induces a variety of tumorigenesis and tumor metastasis and functions as a tumor suppressor (26, 27). miRNAs are regulated by DICER1, and in the other side, many miRNAs have been reported to regulate DICER1. For example, miR-200a inhibited DICER1 expression, therefore attenuating miR-16 maturation, leading to bladder cancer invasion and metastasis (21). miR-122 exerted its prometastatic properties in clear-cell renal cell carcinoma cells by downregulating DICER1 and its downstream effector, the miR-200 family, thereby inducing EMT (17). miR-130a directly targets DICER1 mRNA to enhance SiHa cell (cervical cancer cells) migration and invasion (22). miR-18a promoted growth, invasion of NPC cells by inhibiting DICER1, and impairing miRNA biogenesis (57). These observations including ours proposed the mutually modulated relationships between DICER1 and multiple miRNAs. Although multiple miRNAs could target DICER1, among them, BART2-5p showed significant inhibition effect on DICER1 (Fig. 4*C*), suggesting an impressive role of BART2-5p in the modulation of DICER1-dependent miRNAs homeostasis in NPC cells. We also noted a dynamic negative feedback effect during the inhibition of DICER1 by BART2-5p (Fig. S7). Thus, DICER1 expression reverses the effect of BART2-5p, which may simply accelerate the aforementioned negative feedback. In NPC clinical specimens, BART2-5p was negatively correlated with DICER1 (Fig. 6, *C* and *D*), providing evidence for future translational studying.

The synthesis of intracellular miRNAs is tightly controlled in both time and space (1). The development of human tumors is associated with altered miRNAs expression (3, 4), and overall miRNAs expression levels are reduced in many types of tumor (5, 6). In addition to affecting the gene expression profile of NPC cells, BART2-5p also has regulatory effects on many cellular miRNAs, including upregulation of multiple pro-oncogenic miRNAs expression and downregulation of oncogenic miRNAs. BART2-5p may indiscriminately downregulate the expression of most miRNAs by suppressing DICER1. Considering that there is not a one-to-one correspondence between miRNAs and target genes, it is certain that BART2-5p can function as an oncogene through multiple pathways. Therefore, the study of BART2-5p-DICER1 axis is of great importance for EBV-associated malignance.

BART2-5p is highly correlated with tumor metastasis and also exists in a high level in the serum of the NPC patients, especially with feature of metastasis (9, 52). It is reasonable to speculate that the high amount of BART2-5p in the serum could serve as a predictive biomarker as well as therapeutics target, which is a topic worthy of future attention. Many drugs and therapeutic modalities have been developed for EBV-associated cancers today, but therapeutic options for EBV-positive recurrent or metastatic disease are limited (58, 59). Whether miRNA BART2-5p can become an important therapeutics target regarding NPC metastasis and whether inhibitors against miRNA BART2-5p can be developed deserve high attention.

## Experimental procedures

### Cell culture

EBV-negative NPC cell lines 5-8F, 6-10B, HONE1, and HK1 and EBV-positive NPC cell line HONE1-EBV, and immortalized nasopharyngeal epithelial cell line NP69 were cultured in 1640 medium (Gibco) supplemented with 10% fetal bovine serum. Human renal epithelial cell line derived strain 293T cells were cultured in Dulbecco's modified Eagle's medium (Gibco) supplemented with 10% fetal bovine serum. All cells were maintained at 37 °C and 5% CO_2_.

### Patient samples

Formalin-fixed paraffin-embedded NPC specimens (see Table S3 for patient information) were obtained from the Second Xiangya Hospital of Central South University. Written informed consent was obtained from all study participants. The collection and use of tissue samples were approved by the ethical review committee of the appropriate institution.

### RNA oligos and cell transfection

All miRNA mimics and inhibitors were synthesized by GenePharma Inc. The sequences of BART2-5p mimics were 5′-UAUUCUGCAUUCGCCCUUGC-3' (sense) and 5'- AAGGGCGAAUGCAGAAAAUAUAUU-3' (antisense); the sequences of hsa-miRNA-122 mimics are 5′-UGGAGUGUGACAAUGGUGUUUG-3' (sense) and 5′-AUCGUUACCAGACAGUGIUAUU-3' (antisense); the sequences of hsa-miR-200a mimics are 5′-UAACACUCUGUGGUAACGAUGU-3 ' (sense) and 5′-AUCGUUACCAGACAGUGUUAUU-3' (antisense); the sequence of hsa-miR-130a mimics is 5'- CAGUGCAAUGUUAAAAGGGCAU-3' (sense) and 5′-GCCCUUUUAACAUUGCACUGUUU-3' (antisense); the sequence of the NC mimics was 5 '-UUCUCCGAACGUGUCACGUTT-3' (sense) and 5′-ACGUGACACGUUCGGAGAATT-3' (antisense); the sequence of BART2-5p inhibitor with the sequence 5′-GCAAGGGCGAAUGCAGAAAAUA-3' and the sequence of the NC inhibitor was 5′-CAGUACUUUUGUGUAGUACAA-3'. The target sequence of si1-DICER1 was 5′-AAGGCTTACCTTCTCCAGGCT-3' and the target sequence of si2-DICER1 was 5′-AATTGGCTTCCTCCTGGTTAT-3' (60). Cells were transfected with RNAs and/or plasmids using Lipofectamine 3000 (Invitrogen).

### Lentivirus transduction

The lentiviral packaging system was produced by GenePharma Inc. Stable cell lines were constructed using the lentiviral vector plasmid LV3 (H1/GFP & Puro) in this study. A random flanking sequence control (mock) and EBV-miRNA-BART2-5p were purchased from GenePharma Inc and transduced into cells according to the manufacturer's instructions.

### RT and real-time PCR

Total RNA was extracted using TRIzol reagent (Invitrogen). For mRNA reverse transcription, 2 μg RNA was reversed to complementary DNA (cDNA) using RevertAid First Strand cDNA Synthesis Kit (Thermo Fisher Scientific) according to the manufacturer's protocol. For miRNAs reverse transcription, Mir-X miRNA First Strand Synthesis Kit (Takara), 2 μg RNA was reversed into cDNA according to the manufacturer's protocol. RT-qPCR was performed using specific primers and SYBR Premix Ex TaqII kit (Takara). Expression levels of mRNA and miRNA were quantified by measuring the cycle threshold and normalized to *GAPDH* and *U6*, respectively. Data were further normalized to NCs unless otherwise stated. Primers used for RT-qPCR are shown in Table S2.

### RNA sequencing and miRNA sequencing

6-10B cells were transfected with BART2-5p mimics (or NC mimics) for 48 h. RNA was extracted with mirVana miRNA Isolation Kit (Ambion) and sequenced with the illumina NovaSeq 6000System (Oebiotech). Data extraction and normalization were performed according to the manufacturer's standard protocol. The original expression files and details of RNA-seq and miRNA-seq have been deposited at NCBI Gene Expression Omnibus under the accession number GSE220166.

### Prediction of miRNA target genes

Potential target genes of BART2-5p were predicted by Targetscan Custom (http://www.targetscan.org/vert_50/seedmatch.html) and Tarbase (https://dianalab.e-ce.uth.gr/html/diana/web/index.php?r=tarbasev8) for prediction, followed by taking the intersection of the predicted genes with those significantly downregulated genes by BART2-5p in RNA-seq to obtain them.

### *In vitro* migration and invasion assays

Transwell assay was performed to evaluate cell migration and invasion. Each transwell was seeded with 30,000∼50,000 NPC cells/200 μl of serum-free medium (for invasion assay, 50 μl of diluted stromal gel was added, and the dilution ratio was stromal gel: serum-free medium = 1:8). The process was terminated with 4% paraformaldehyde fixation and crystalline violet staining, and the cells were gently washed away from the inside of the chambers with cotton swabs and photographed under the microscope for counting.

### Cell proliferation assay

Cell counting kit-8 assays were performed according to the manufacturer's instructions (APExBIO). Briefly, NPC cells were inoculated in 96-well plates at a density of 1000 cells/well. At 24, 48, 72, and 96 h later, 10 μl of CCK8 solution was added to each well of the plate and the wells were incubated for 2.5 h. The absorbance at 450 nm was assessed using a multifunctional enzyme marker.

### Western blotting

Cells were collected and lysed with RIPA buffer (Beyotime) supplemented with protease inhibitor and phosphatase inhibitor (Bimake). Proteins were separated by SDS-PAGE and transferred to polyvinylidene difluoride membranes (Millipore). The membranes were incubated overnight at 4 °C with the primary antibodies listed in Table S1, and then horseradish peroxidase-coupled secondary antibodies were performed at 37 °C for 1 h. The antigen–antibody reaction was observed by enhanced chemiluminescence.

### Flow cytometry assays (cell cycle or apoptosis)

Cell cycle progression assay (Cell Cycle and Apoptosis Analysis Kit, Beyotime) and apoptosis detection (Annexin V-FITC/PI Apoptosis Detection Ki, Vazyme) were performed according to the manufacturer's instructions, and finally cells were detected and quantitatively stained by flow cytometry.

### Plasmid and dual-luciferase reporter analysis

The potential target WT sequence and MUT sequence on DICER1 3′-UTR were subcloned into pmirGLO dual luciferase miRNA expression vector, which was synthesized and constructed by GenScript. To validate the miRNA target genes, 293T cells were seeded into 12-well plates and co-transfected with 500 ng of luciferase reporter gene plasmids (DICER1 3′UTR WT or MUT) and 40 pmol BART2-5p or NC mimics. Firefly and Renilla luciferase activities were measured by the Dual-Glo luciferase assay system (Vazyme). Data are expressed as relative firefly luciferase activity normalized to Renilla luciferase values.

### Immunohistochemistry and RNA *in situ* hybridization staining

Immunohistochemical staining of paraffin sections of NPC clinical specimens was performed to determine DICER1 protein levels. Briefly, tumor tissue embedded in paraffin was dewaxed with xylene and hydrated in ethanol. The slides were placed in boiling sodium citrate buffer (10 mmol/L, pH 6.0) for 5 min to repair the antigen. Three percent H_2_O_2_ blocked the endogenous peroxidase activity of the tumor tissue. After incubation with goat serum to block nonspecific binding sites, the tumor was incubated with anti-DICER1 antibody (Cell Signaling Technology) at 4 °C overnight. Biotinylated goat anti-rabbit IgG antibody was then added and incubated. Paraffin sections of clinical specimens were subjected to EBV-miRNA-BART2-5p *in situ* hybridization assay using the EBV-miRNA-BART2-5p probe ISH kit (Boster). Finally, all sections were incubated with enzyme-labeled streptavidin and then developed with 3,3-diaminobenzidine solution. After restaining with hematoxylin, the sections were dehydrated and sealed. Depending on the intensity and number of cells stained, they were divided into four levels, with 0 indicating no positive staining (negative), one indicating light yellow (weakly positive), two indicating brown (positive), and three indicating brown (strongly positive).

### RNA immunoprecipitation assays

Cells were collected and washed twice in prechilled PBS. Cell pellets were resuspended in mild lysis buffer and incubated on ice for 30 min. After centrifugation at 12,000 rpm for 15 min at 4 °C, the supernatant was incubated with anti-Ago2 antibody (HuaBio) or control homologous anti-IgG antibody attached to protein A agarose beads, spun for 4 h at 4 °C, and then RNA was extracted and analyzed by RT-qPCR.

### *In vivo* metastasis assay

The BALB/C male nude mice used in this experiment were purchased from Hunan SJA Laboratory Animal Co, Ltd and weighed 15 ± 2 g. They were all quality checked and housed in the sun protection factor environment of the Department of Laboratory Animal Science, Hunan University of Traditional Chinese Medicine. The nude mice were randomly divided into three groups at 6 to 8 weeks of age: NC group (injected with 6–10B cells with GFP-stable expression transfected with NC mimics and blank vector), B2-5p group (injected with cells transfected with BART2-5p mimics and blank vector) and B2-5p+DICER1 group (injected with cells transfected with BART2-5p mimics and DICER1 expression plasmid). A total of 2 × 10^6^ 6-10B cells were suspended in 200 ml serum-free RPMI1640 medium and injected into the tail vein of nude mice. The status of mice was checked twice a week and all deaths were recorded. After eight weeks, the remaining nude mice were sacrificed for GFP imaging and the fluorescence intensity was quantified using Living Image software (PerkinElmer). Finally, major organs were fixed in paraformaldehyde for H&E staining.

### Characterization experiments of the function of DICER1 RNase endonuclease cleaving stem-loop double-stranded RNA

DICER1 enzyme function was characterized using 5-8F cells stably expressing LV3-mock (H1/GFP & Puro). The experiments were divided into four groups: (1) co-transfection of NC mimics and scramble plasmids; (2) co-transfection of NC mimics and pLKO.1 GFP shRNA; (3) co-transfection of BART2-5p mimics and scramble plasmids; (4) co-transfected with BART2-5p mimics and pLKO.1 GFP shRNA. The expression of GFP was observed under the microscope 48 h after transfection as a readout of DICER1 RNase endonuclease enzyme function.

### Statistical analysis

Statistical analysis was completed using SPSS 26.0 and GraphPad Prism 8 statistical software (https://lib.csu.edu.cn/info/1598/2865.htm). The *t* test was used to compare the means of measurement data between two groups; the analysis of variance one way (ANOVA) was used to compare the means of multiple samples of measurement data. *p* < 0.05 is considered statistically significant.

## Data availability

All data are contained in the manuscript, except for the sequencing data, which has been saved to the Gene Expression Omnibus database under the accession number GSE220166.

## Supporting information

This article contains supporting information.

## Conflict of interest

The authors declare that they have no conflicts of interest with the contents of this article.
