# Supplementary material for: Epstein–Barr virus microRNA miR-BART2-5p accelerates nasopharyngeal carcinoma metastasis by suppressing RNase Ⅲ endonuclease DICER1
Source: J Biol Chem. 2023 Jul 24;299(9):105082. doi: 10.1016/j.jbc.2023.105082 (PMC10470218; doi:10.1016/j.jbc.2023.105082)
Supplement: Supporting Figures S1–S9 [file mmc1.pdf]

Supplementary Table1. Antibodies list

Antibody	Source	Catalogue
DICER1	Cell Signaling Tech.	5362T
GAPDH	Proteintech, China	60004-1-Ig
Cadherin-1	ZENBIO, China	340341
Cadherin-2	ZENBIO, China	380671
Vimentin	ZENBIO, China	R22775
Fibronectin	HuaBio, China	JF0582
β-catenin	Cell Signaling Tech.	8480S
C/EBPβ	HuaBio, China	SC55-05
MAPRE1	Cloud-Clone, China	MAG491Hu25
Argonaute 2 (Ago2)	HuaBio, China	JF0992
isotype control rabbit IgG for RIP assay	Proteintech, China	30000-0-AP
HRP-conjugated Affinipure Goat IgG(H+L)	Proteintech, China	SA00001-1 and SA00001-2

Supplementary Table 2. Primers sequences used for qRT-PCR

Genes	Sequences（5'-3'）	
	Forward	Reverse
DICER1	TGCTATGTCGCCTTGAATGTT	AATTTCTCGATAGGGGTGGTCTA
CDH1	TTCTGGAAGGAATGGAGGAGTC	ACCTGGAATTGGGCAAATGTG
CDH2	AGCCAACCTTAACTGAGGAGT	GGCAAGTTGATTGGAGGGATG
VIM	AGATGGCCCTTGACATTGAG	TGGAAGAGGCAGAGAAATCC
FN1	GGTGACACTTATGAGCGTCCTAAA	AACATGTAACCACCAGTCTCATGTG
SNAI1	GCTGCAGGACTCTAATCCAGAGTT	GACAGAGTCCCAGATGAGCATTG
SNAI2	TGTGACAAGGAATATGTGAGCC	TGAGCCCTCAGATTTGACCTG
CD44	CTGCCGCTTTGCAGGTGTA	CATTGTGGGCAAGGTGCTATT
ZEB1	GCACAACCAAGTGCAGAAGA	GCCTGGTTCAGGAGAAGATG
ZEB2	CAAGAGGCGCAAACAAGCC	GCCTGGTTCAGGAGAAGATG
MMP2	CAACTACGATGATGACCGCAA	GTGTAAATGGGTGCCATCAGG
CTNNB1	TCACCAACTGGGACGACATG	GTCACCGGAGTCCATCACGAT
CEBPB	CGTGGACAAGCACAGCGACGAG	CTTGAACAAGTTCCGCAGGGTG
MAPRE1	AAGCTAGAACACGAGTACATCCA	AGTTTCTTGACCTTGTCTGGC
miR-16-2-3p	CGCGCCAATATTACTGTGCT	Universal stem-loop Reverse Primer (Vazyme)
miR-23a-5p	GGGGGTTCCTGGGGATG	Universal stem-loop Reverse Primer (Vazyme)
let-7c-3p	GCGCGCTGTACAACCTTCTAG	Universal stem-loop Reverse Primer (Vazyme)
GAPDH	GGAGCGAGATCCCTCCAAAAT	GGCTGTTGTCATACTTCTCATGG
EBV-miR-BART2-5p	TATTTTCTGCATTCGCCCTTGC	Universal Reverse Primer (Takara)
miR-122-3p	AACGCCATTATCACACTAAATA	Universal Reverse Primer (Takara)
miR-130a-3p	CAGTGCAATGTTAAAAGGGCAT	Universal Reverse Primer (Takara)
miR-200a-3p	TAACACTGTCTGGTAACGATGT	Universal Reverse Primer (Takara)
U6	Provided by Mir-X miRNA First-Strand synthesis kit (Takara)	Provided by Mir-X miRNA First-Strand synthesis kit (Takara)

Supplementary Table 3. The clinicopathological parameters of patients

Variable	Number of cases
**Age (years)**	
≥50	12
<50	14
**Gender**	
Male	17
Female	9
**Disease status**	
Nasal polyp	8
Poorly differentiated carcinoma	18
