# Supplementary material for: Epstein–Barr virus microRNA miR-BART2-5p accelerates nasopharyngeal carcinoma metastasis by suppressing RNase Ⅲ endonuclease DICER1
Source: J Biol Chem. 2023 Jul 24;299(9):105082. doi: 10.1016/j.jbc.2023.105082 (PMC10470218; doi:10.1016/j.jbc.2023.105082)

Epstein–Barr virus microRNA miR-BART2-5p accelerates nasopharyngeal carcinoma metastasis by suppressing RNase III endonuclease DICER1

Supplementary Figures

Supplementary Figure 1

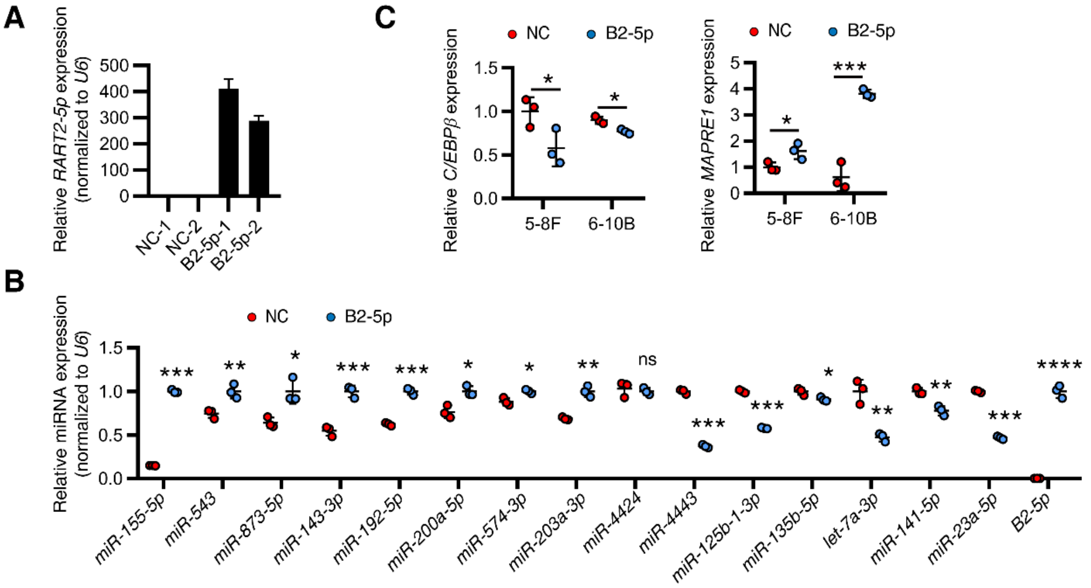

Supplementary Figure 1. BART2-5p modulates multiple microRNAs' expressions in NPC cells

(A) BART2-5p mimics or the corresponding negative control (NC) mimics were transfected into 6-10B cells (two replicates per group), then, RNA was collected. RNA sequencing was performed to analyze the effect of BART2-5p on the gene/microRNA expression profile of 6-10B cells (see Fig.2A, 3A). RT-qPCR experiments demonstrated that BART2-5p was successfully expressed in 6-10B cells. *U6* was used for normalizing the expression of BART2-5p.

(B) RT-qPCR was employed to assay the impact of BART2-5p mimics on host microRNAs in 6-10B cells. Those host microRNAs were selected from the microRNA-seq results (see Fig.3A, B).

(C) BART2-5p mimics or NC mimics were transfected into 5-8F cells and 6-10B cells. RT-qPCR was employed to detect the mRNA level of *C/EBPβ* and *MAPRE1.*

Supplementary Figure 2

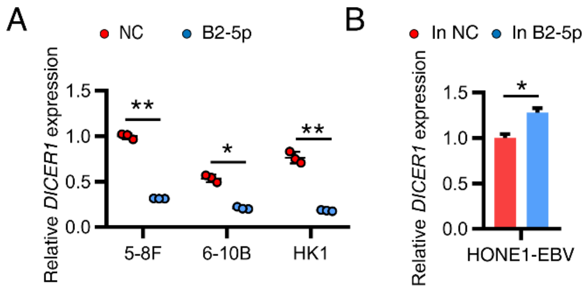

Supplementary Figure 2. BART2-5p suppresses *DICER1* mRNA expression in NPC cells

(A) BART2-5p mimics or negative control (NC) mimics were transfected into NPC cells and the *DICER1* mRNA expression levels were assayed by RT-qPCR.

(B) BART2-5p inhibitors or negative control (NC) inhibitors were transfected into HONE1-EBV cells and the *DICER1* mRNA expression levels were assayed by RT-qPCR. (In: inhibitor)

Supplementary Figure 3

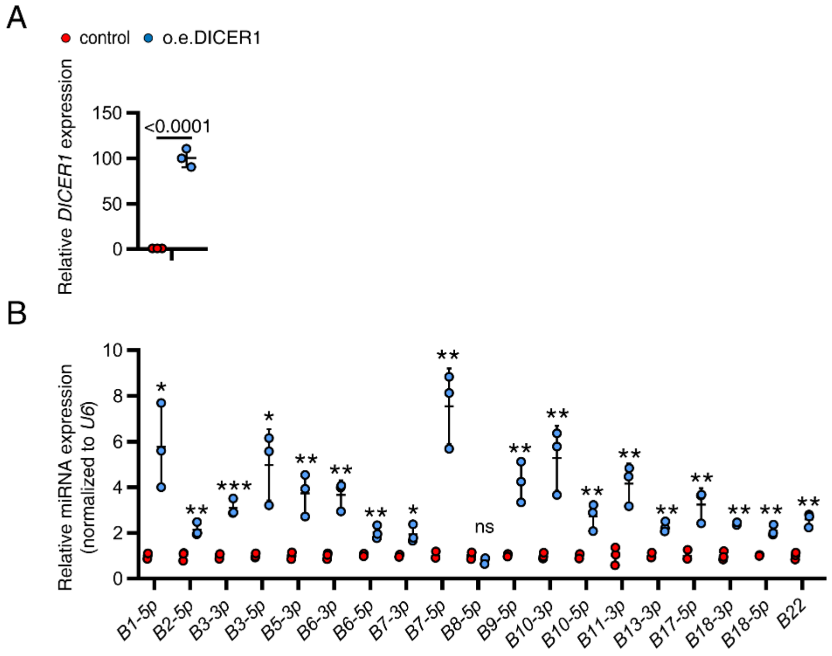

Supplementary Figure 3. DICER1 indiscriminately increases the expression of EBV-encoded microRNAs

(A) DICER1 or negative control expression vectors were transfected into HONE1-EBV cells, and the *DICER1* mRNA expression levels were assayed by RT-qPCR.

(B) DICER1 or negative control expression vectors were transfected into HONE1-EBV cells, and the indicated EBV-encoded microRNAs expression levels were assayed by RT-qPCR.

Supplementary Figure 4

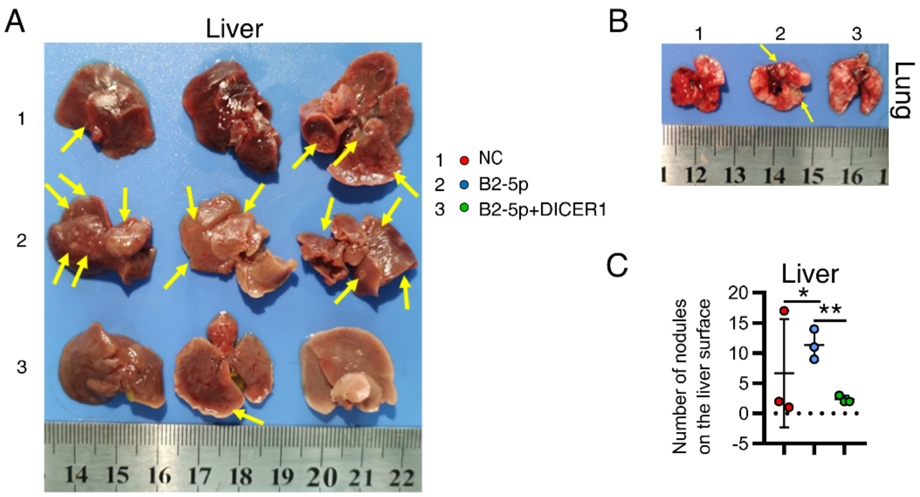

Supplementary Figure 4. Surface nodules of lung and liver tissue in the tail vein metastasis nude mice model. (Supplementary to Fig. 5B, C)

Supplementary Figure 5

A

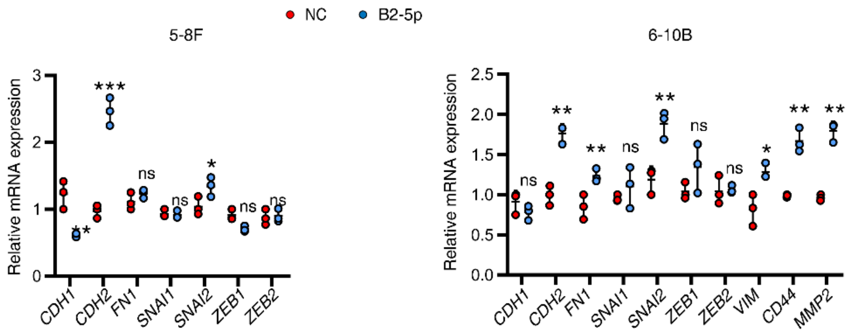

B

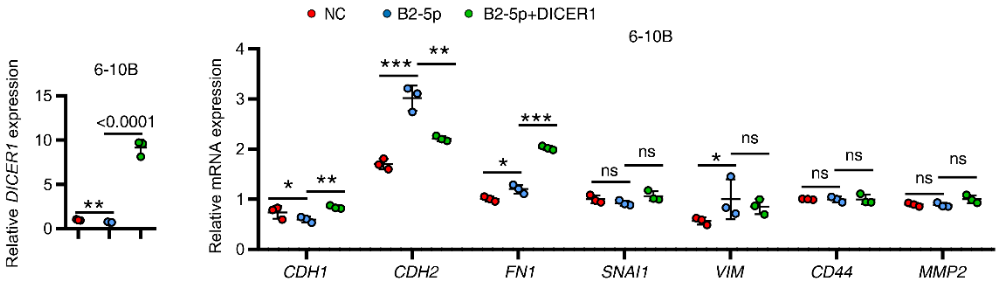

Supplementary Figure 5. BART2-5p modulates EMT-related molecules expressions.

(A) BART2-5p mimics or negative control (NC) mimics were transfected into NPC cells and the indicated EMT-related molecules expression levels were assayed by RT-qPCR.

(B) In the BART2-5p transfected 6-10B cells, DICER1 expression vectors were re-expressed, and the EMT-related molecules expression levels were assayed by RT-qPCR.

Supplementary Figure 6

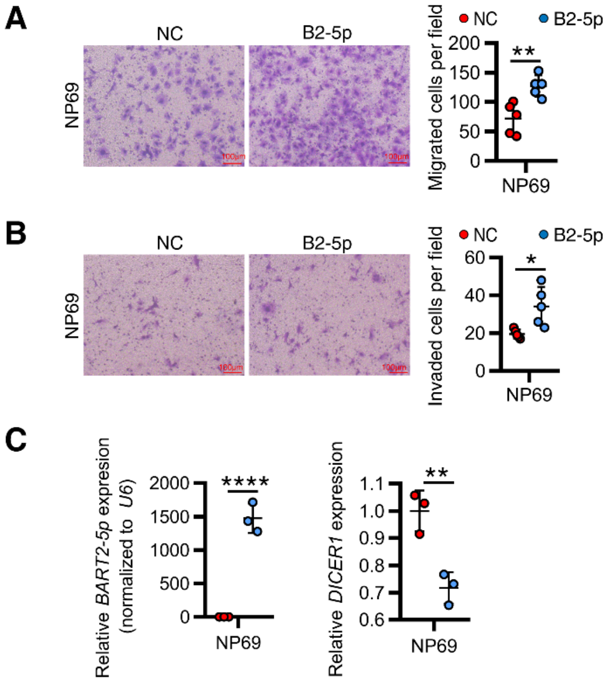

Supplementary Figure 6. BART2-5p promotes the migration and invasion of normal nasopharyngeal epithelial cell lines NP69 in vitro. (A) After transfection of BART2-5p mimics and NC mimics into NP69 cells for 48 h (n=5). 40,000 cells from each group were inoculated in transwell chambers and transwell migration assays were performed with representative images on the left and statistical plots of migrating cell numbers on the right. (B) After transfection of BART2-5p mimics and NC mimics into NP69 cells for 60 h (n=5). 40,000 cells from each group were inoculated in transwell chambers and transwell invasion assays were performed with representative images on the left and statistical plots of invading cell numbers on the right. (C) The transfection efficiency of BART2-5p mimics and the *DICER1* mRNA expression levels were assayed by RT-qPCR.

Supplementary Figure 7

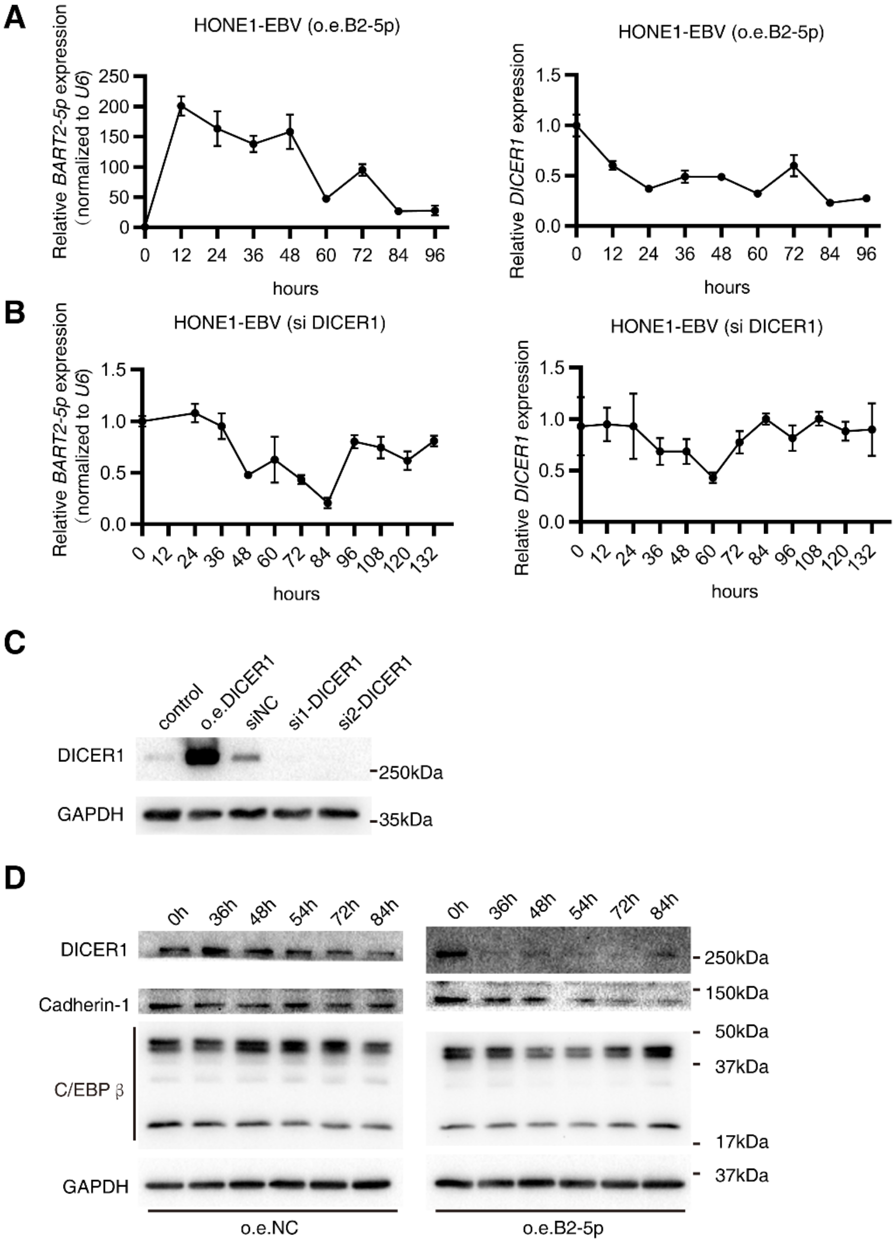

Supplementary Figure 7. Dynamic negative feedback effect during the inhibition of DICER1 by BART2-5p. (A) Upon transfection of BART2-5p mimics into HONE1-EBV cells, mRNA levels of BART2-5p and DICER1 were detected at 0 h, 12 h, 24 h, 36 h, 48 h, 60 h, 72 h, 84 h and 96 h post-transfection by RT-qPCR. (B) Upon transfection of siRNA-DICER1(si DICER1) into HONE1-EBV cells, mRNA levels of BART2-5p and DICER1 were detected at 0 h, 12 h, 24 h, 36 h, 48 h, 60 h, 72 h, 84 h, 96 h, 108 h,120 h, and 132 h post-transfection by RT-qPCR. (C) The transfection efficiency of siRNA-DICER1 and DICER1 expression plasmid was detected by Western blotting. (D) Upon transfection of BART2-5p mimics (or NC mimics) into HONE1-EBV cells, the protein levels of DICER1, C/EBPβ, and Cadherin-1 were detected by Western blotting.

Supplementary Figure 8

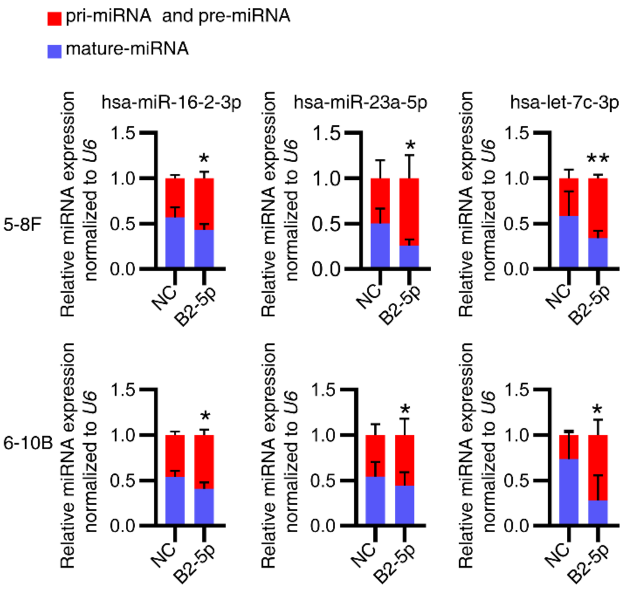

Supplemental Figure 8. BART2-5p delays the transition of pre-miRNA to mature-miRNA. After transfection of BART2-5p mimics (or NC mimics) into 5-8F cells or 6-10B cells for 48 h, RT-qPCR was performed to detect the overall levels of pri-miRNA and pre-miRNA using the primers as described (Ref. Real-time expression profiling of microRNA precursors in human cancer cell lines, Nucleic Acids Res. 2005;33(17):5394-403). The stem-loop method was used to detect mature microRNAs. The overall expression levels of mature microRNAs and corresponding pri-miRNA and pre-miRNA were then set as 1. The percentage of mature microRNAs and corresponding pri-miRNA and pre-miRNA expression was displayed in a bar stacked graph. Data are shown as the means ± S.D. . Statistical significance relative to control was assessed by the unpaired two-tailed Student's t test. *, $p<0.05$; **, $p<0.01$ compared with the control group.

Supplementary Figure 9

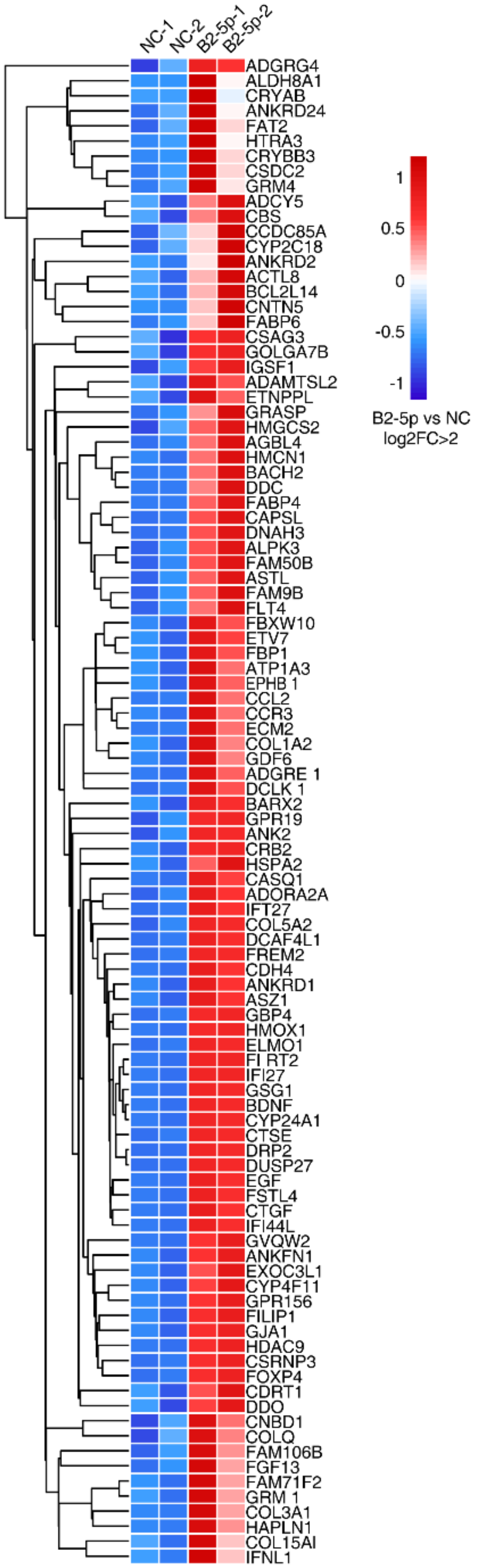

Supplementary Figure 9. Heatmap showing log2(FC)>2 (4-fold upregulated) genes for mRNA-seq (B2-5p mimics vs NC mimics).

Original blots of western blots

Supplementary to Fig.3D

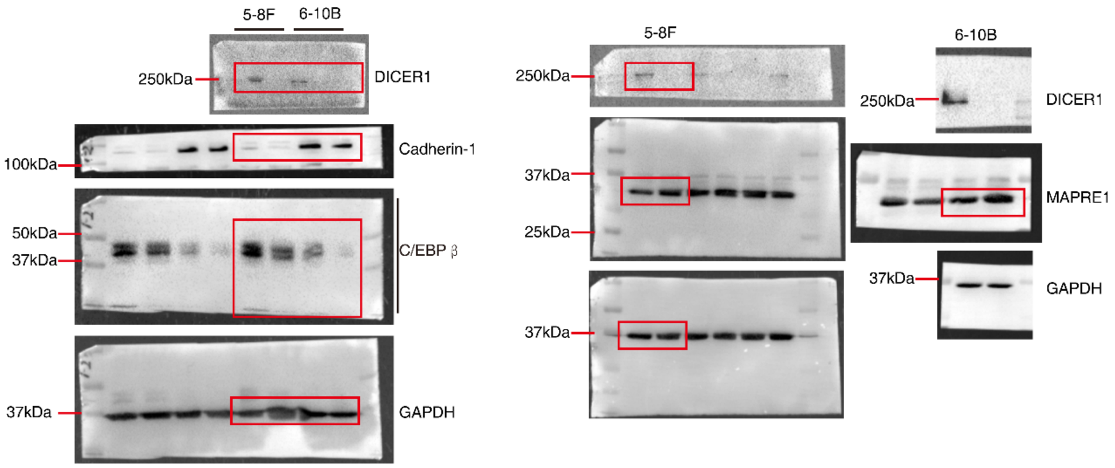

Supplementary to Fig.4B

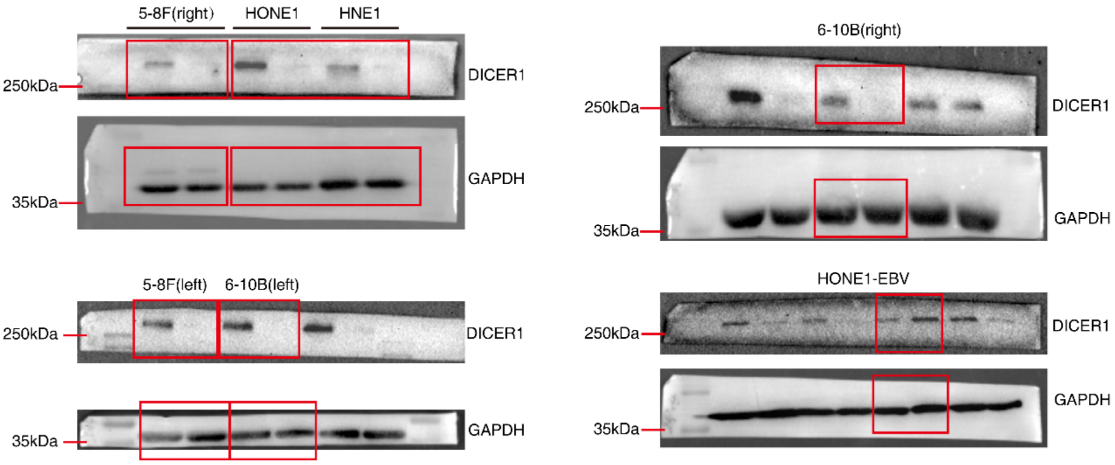

Supplementary to Fig.4C

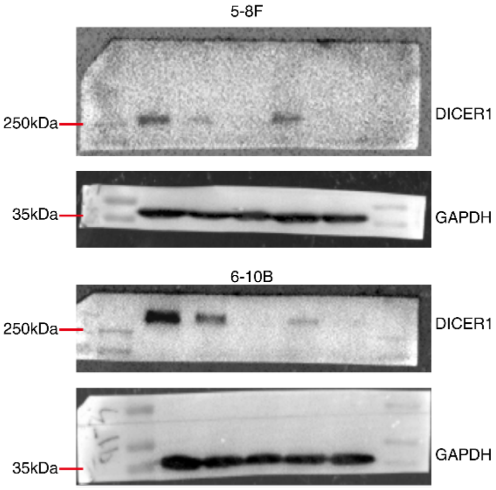

Supplementary to Fig.5E

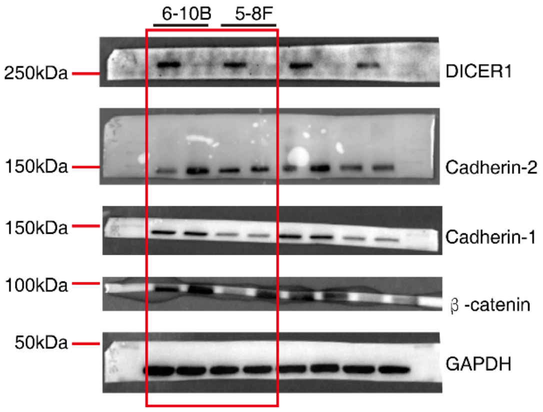

Supplementary to Fig.5F

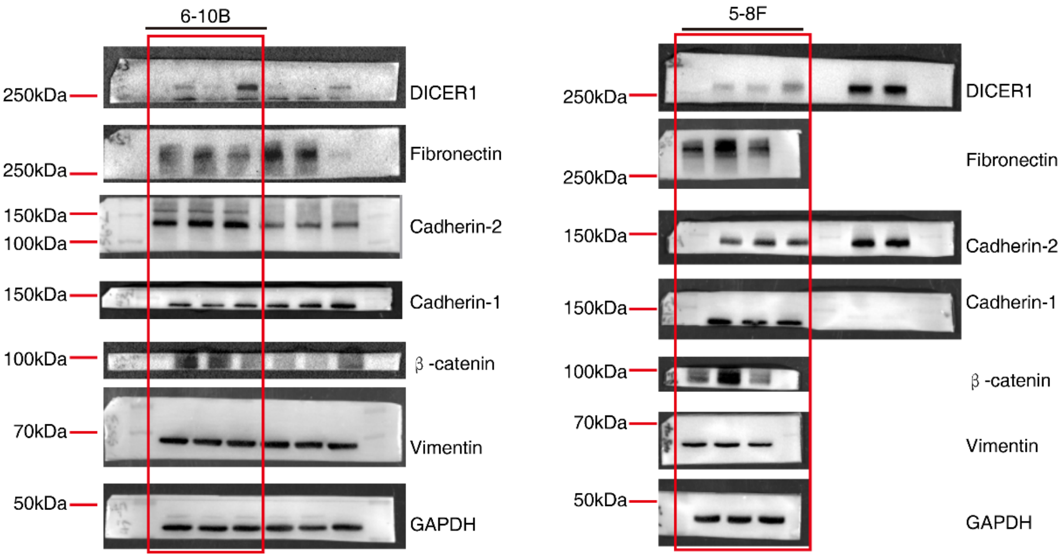

Supplementary to Fig.S7D

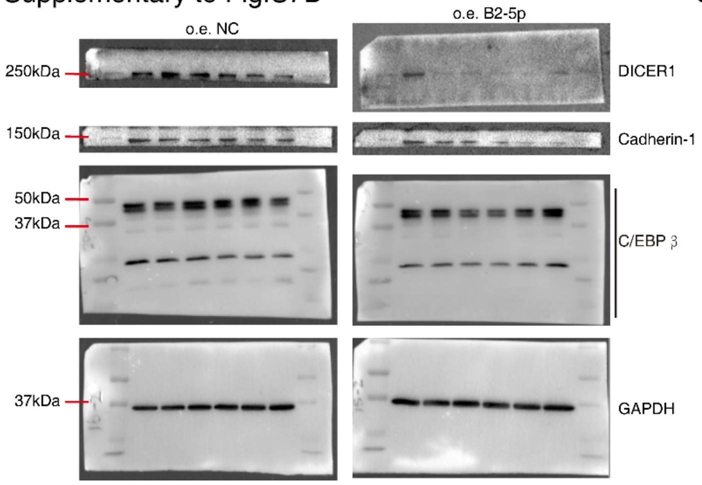

Supplementary to Fig.S7C

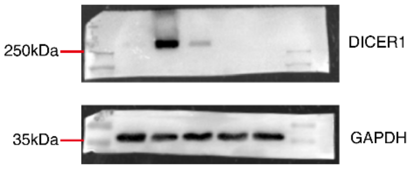

Supplement: Supporting Tables S1–S3 [file mmc2.pdf]
